# *Bifidobacterium longum* counters the effects of obesity: Partial successful translation from rodent to human

**DOI:** 10.1016/j.ebiom.2020.103176

**Published:** 2020-12-18

**Authors:** Harriët Schellekens, Cristina Torres-Fuentes, Marcel van de Wouw, Caitriona M. Long-Smith, Avery Mitchell, Conall Strain, Kirsten Berding, Thomaz F.S. Bastiaanssen, Kieran Rea, Anna V. Golubeva, Silvia Arboleya, Mathieu Verpaalen, Matteo M. Pusceddu, Amy Murphy, Fiona Fouhy, Kiera Murphy, Paul Ross, Bernard L. Roy, Catherine Stanton, Timothy G. Dinan, John F. Cryan

**Affiliations:** a APC Microbiome Ireland, University College Cork, Cork, Ireland; b Department of Anatomy and Neuroscience, University College Cork, Cork, Ireland; c Teagasc Food Research Centre, Moorepark, Fermoy, Cork, Ireland; d College of Science Engineering & Food Science, University College Cork, Cork, Ireland; e Cremo SA, Villars-sur-Glâne, Fribourg, Switzerland; f Dept of Psychiatry and Behavioural Neuroscience, University College Cork, Cork, Ireland

**Keywords:** Obesity, Translational, Fasting blood glucose, Ghrelin, Cortisol, Gut microbiota, Probiotic, *Bifidobacterium longum*

## Abstract

**Background:**

The human gut microbiota has emerged as a key factor in the development of obesity. Certain probiotic strains have shown anti-obesity effects. The objective of this study was to investigate whether *Bifidobacterium longum* APC1472 has anti-obesity effects in high-fat diet (HFD)-induced obese mice and whether *B. longum* APC1472 supplementation reduces body-mass index (BMI) in healthy overweight/obese individuals as the primary outcome. *B. longum* APC1472 effects on waist-to-hip ratio (W/H ratio) and on obesity-associated plasma biomarkers were analysed as secondary outcomes.

**Methods:**

*B. longum* APC1472 was administered to HFD-fed C57BL/6 mice in drinking water for 16 weeks. In the human intervention trial, participants received *B. longum* APC1472 or placebo supplementation for 12 weeks, during which primary and secondary outcomes were measured at the beginning and end of the intervention.

**Findings:**

*B. longum* APC1472 supplementation was associated with decreased bodyweight, fat depots accumulation and increased glucose tolerance in HFD-fed mice. While, in healthy overweight/obese adults, the supplementation of *B. longum* APC1472 strain did not change primary outcomes of BMI (0.03, 95% CI [-0.4, 0.3]) or W/H ratio (0.003, 95% CI [-0.01, 0.01]), a positive effect on the secondary outcome of fasting blood glucose levels was found (-0.299, 95% CI [-0.44, -0.09]).

**Interpretation:**

This study shows a positive translational effect of *B. longum* APC1472 on fasting blood glucose from a preclinical mouse model of obesity to a human intervention study in otherwise healthy overweight and obese individuals. This highlights the promising potential of *B. longum* APC1472 to be developed as a valuable supplement in reducing specific markers of obesity.

**Funding:**

This research was funded in part by Science Foundation Ireland in the form of a Research Centre grant (SFI/12/RC/2273) to APC Microbiome Ireland and by a research grant from Cremo S.A.

**Research in context**

**Evidence before this study**

• Evidence has shown that the gut microbiota is an important component in the regulation of the host's physiology and metabolism, modulating energy harvest, storage and expenditure and, therefore, represents a promising target in the treatment of obesity and obesity-related disorders.
• Different probiotic strains have been shown to have different beneficial anti-obesity effects such as reduced body weight gain, improvements in insulin sensitivity and glucose uptake, and reduced fat depots accumulation in rodents.
• The *Bifidobacterium longum* APC1472 strain was recently identified in our laboratory to modulate ghrelinergic signalling *in vitro*, which is an important signalling pathway modulating central appetite regulation and metabolism.

**Added value of this study**

• *B. longum* APC1472 demonstrated several significant beneficial effects in HFD-induced obese mice.
• *B. longum* APC1472 reduced fasting glucose, cortisol awakening responses and increased active ghrelin in healthy obese adults.
• Effects of *B. longum* APC1472 partially translated from a preclinical mouse model to a human intervention study where this probiotic positively impacted markers of obesity.

**Implications of available evidence**

• *B. longum* APC1472 has promising potential to be developed as a valuable supplement in reducing specific markers of obesity and is poised to have significant relevance in conditions of heightened blood glucose, such as type 2 diabetes.

Alt-text: Unlabelled box

## 1 Introduction

Obesity is one of the most pervasive, chronic diseases globally, in both developed and developing countries, contributing to at least 2.8 million deaths annually and significantly impacting the healthcare system [1]. The growing obesity epidemic is associated with increases in several comorbidities, such as cardiovascular disease, stroke, metabolic syndrome, type 2 diabetes and cancer [2,3]. Current available anti-obesity therapeutics are limited and associated with poor efficacy and adverse side effects [4,5]. Diet and exercise have been demonstrated to be the most potent in reducing obesity symptomatology [6]. In addition, natural compounds and their derivatives have been proposed as safer anti-obesity alternatives, either as functional foods or nutraceuticals [4].

The gut microbiota has emerged as a key component in the development of obesity and modulates the host's physiology and metabolism, including energy harvest, storage and expenditure [4,[7], [8], [9], [10], [11], [12], [13]]. Preclinical and clinical evidence demonstrating the critical role of the gastrointestinal microbiota on host metabolism is steadily increasing. For example, germ-free mice are protected against obesity and are significantly leaner than normal control mice despite consuming more calories [14]. In addition, faecal transplantation from obese donors was shown to replicate the obese phenotype in lean germ-free mice independent of diet [15], [16], [17]. Moreover, accelerated post-dieting weight regain is associated with a persistent intestinal microbiome signature after successful dieting in obese mice [18].

Nonetheless, the exact mechanisms of how diet-induced changes in gut microbiota affect gut-brain signalling, including host metabolism, appetite regulation and brain health, are currently still lacking [19,20]. Interestingly, the obese-associated microbiota has been shown to have an increased capability to harvest energy from food and contributes to host insulin resistance, gut permeability, low-grade inflammation, and fat deposition [21,22]. Intestinal microbiota-derived metabolites have also been shown to impact the central regulation of appetite [9,23,24]. For example, certain bacterial strains modify gut peptides secretion, such as glucagon-like peptide (GLP)−1, thus contributing to hypothalamic appetite and satiety signalling via afferent nerve fibres of the vagus nerve as well as by direct secretion into the circulatory system [24,25]. Furthermore, germ-free mice display marked decreases in expression of intestinal satiety peptides, including cholecystokinin (CCK), peptide tyrosine-tyrosine (PYY) and GLP-1 and also lower circulating levels of leptin and ghrelin [26]. In addition, serum ghrelin levels are negatively correlated with the abundance of certain bacterial taxa, including *Bifidobacterium* and *Lactobacillus* species [27]. Moreover, intake of the prebiotic oligofructose, which promotes the growth of *Bifidobacterium* and *Lactobacillus*, decreases the secretion of ghrelin in obese humans [28]. Taken together, modulation of the gut microbiota is emerging as a promising strategy for the management of obesity and obesity-related disorders such as type-2 diabetes and cardiovascular disease [4,[7], [8], [9],^29^].

Several probiotic strains with different anti-obesity effects in humans have been identified [4,[30], [31], [32], [33], [34], [35], [36]]. The bacterial strain *B. longum* APC1472 has recently been shown to modulate ghrelinergic signalling *in vitro* [37], highlighting the therapeutic potential for host metabolism, appetite and obesity modulation. The ghrelin receptor (GHS-R1a) is activated by the endogenous hormone ghrelin, the first and only known peripheral orexigenic peptide, which regulates peripheral metabolism and energy expenditure as well as centrally regulated homeostatic appetite and food-motivated reward signalling, governing eating behaviour and food intake [38], [39], [40], [41], [42]. Interestingly, obese individuals have attenuated postprandial suppression of ghrelin and a blunted nocturnal plasma ghrelin increase, reinforcing aberrant ghrelinergic signalling in obesity [43,44]. While the precise site of action of ghrelin is somewhat controversial [45], [46], [47], the high prevalence of the ghrelin receptor throughout the small and large intestine, make it a likely target for interaction with the gut microbiota and thus may hold potential as a local therapeutic target [48].

As such, we investigated *B. longum* APC1472 for its ability to ameliorate high-fat diet (HFD)-induced obesity in mice and observed significant beneficial beneficial effects on adiposity and metabolism. Based on these promising effects of *B. longum* in the preclinical model, we subsequently investigated whether it could improve obesity symptomatology in healthy overweight/obese adults. The primary objective of the human intervention study was to determine whether a 12-week daily supplementation of *B. longum* APC1472 decreases body-mass index (BMI), while the secondary objective was to investigate the effects on waist-to-hip ration (W/H ratio), and biomarkers associated with obesity, such as glucose, insulin, HbA1c and ghrelin levels. The exploratory objectives were to investigate the impact of *B. longum* APC1472 on the gut microbiota composition and diversity, peripheral inflammatory profile, stress hormone profile, self-reported perceived stress, anxiety and satiety.

## 2 Methods – animal study

### 2.1 Animals, diets and ethical approval

Five-week-old male C57BL/6 mice (Harlan Laboratories, UK) (40 mice, *n* = 8–10 per group) were housed in groups of 2 mice per cage in standard holding cages with free access to food and water in the animal care facility of University College Cork. The holding room temperature (21 ± 1 °C) and humidity (55 ± 10%) were controlled under a 12 h light/dark cycle (lights on 7.00 AM, lights off 7.00 PM). The mice were fed a low-fat diet (LFD) (10% fat (kcal/100 g), D12450B, Research Diet, USA) or a high-fat diet (HFD) (45% fat (kcal/100 g), D12451, Research Diet, USA) for 16 weeks. Food intake was recorded once per week and calculated on the basis of two mice per cage and five cages per group. The data were reported as cumulative food intake per mouse. Bodyweight was monitored weekly for 15 weeks. Experiments were conducted in accordance with the European Directive 86/609/EEC and the Recommendation 2007/526/65/EC and were approved by the Animal Experimentation Ethics Committee of University College Cork.

### 2.2 *In vivo* probiotic administration

*Bifidobacterium longum* APC1472 was grown anaerobically in De Man, Rogosa and Sharpe (MRS) medium as previously described [37]. The bacterial cell pellet was washed and concentrated in sterile phosphate buffered saline (PBS) containing 25% Glycerol (v/v) to an end concentration of ~7.5 × 10^9^ CFU/mL, aliquoted and stored at −80 °C. Aliquots were defrosted daily just prior to the start of the dark phase and diluted to ~2 × 10^8^ CFU/mL in drinking water for administration to LFD-fed and HFD-fed mice for 16 weeks. Water intake was monitored throughout the experiment. Drinking water containing an equivalent end concentration of sterile PBS (2% v/v) and glycerol (0.5% v/v) was administered to control mice. Water was replaced for probiotic/vehicle-free water every morning. *B. longum* APC1472 survival in drinking water (distilled water) in ambient temperature and oxygen content was tested over 24 h prior to the start of the experiment. Bacteria counts (CFU/mL) did not decrease over 1 log unit for the first 12 h suggesting adequate viability of the strain upon the time of consumption (**Figure S1A)**. No significant changes in water intake were observed within the same diet groups (**Figure S1B**).

### 2.3 *In vivo* glucose tolerance test

Glucose tolerance was assessed after 15 weeks of treatment as previously described [49], with minor modifications. Briefly, mice were fasted for 7 h during the light phase, with free access to water. Glucose levels were measured in tail vein blood using a glucometer (Bayer, UK) immediately before and 15, 30, 60, 90 and 120 min after intraperitoneal injection of glucose (1 g/kg of body weight in sterile saline).

### 2.4 Murine tissue sampling

Mice were euthanized by decapitation. Trunk blood was collected in tubes containing 25 μM dipeptidyl peptidase IV (DPP-IV) inhibitor, 2x protease inhibitor cocktail (Roche) (diluted in PBS) and 0.1% Na_2_ EDTA for an expected blood volume of 400 µL, centrifuged at 3500 g for 15 min at 4 °C and placed on dry ice until storage at −80 °C for further analysis. Adipose depots (epididymal, subcutaneous, mesenteric and retroperitoneal) were dissected and weighed. Whole-brains were collected and placed for 8–10 s into ice-cold isopentane. All tissues were frozen on dry ice and subsequently stored at −80 °C for further analysis.

### 2.5 Murine biochemical analysis

Plasma insulin and leptin levels were analysed by ELISA using the MILLIPLEX® MAP Mouse Metabolic Hormone Magnetic Bead Panel (Millipore, MMHMAG-44 K) accordingly to the manufacturer's instructions. Plasma ghrelin levels were analysed using the Rat/Mouse Ghrelin (Total) ELISA Kits (Millipore, EZGRA-88 K). Triglycerides levels were analysed with a Triglyceride Quantification Kit (Abcam Ltd, ab65336) following the to manufacturer's instructions. Corticosterone levels were assayed using ELISA kits (Enzo Life Sciences, ADI-900–097) according to the manufacturer's instructions.

### 2.6 Murine RNA isolation and quantitative real-time PCR

Hypothalamus was dissected with a forceps (macropunch) from the frozen brain on dry ice and immediately processed for RNA extraction. Hypothalamus and epididymal adipose tissue total RNA were extracted using the mirVana™ miRNA Isolation kit (Ambion/Life Technologies, AM1560) and RNeasy® Lipid Tissue Mini Kit (Qiagen, 74,804), respectively with DNase treatment using Turbo DNA-free (Ambion/life Technologies, AM1907) according to the manufacturer's recommendations. Equal amounts of RNA were first reverse transcribed to cDNA using High Capacity cDNA Reverse Transcription Kit (Applied Biosystems, 4,368,814). Real-time PCR was performed using TaqMan Universal Master Mix II, no Uracil-N glycoslyase (UNG) on a LightCycler®480 System (Roche). Mouse β-actin control mix Probe dye: VIC-MGB (Applied Biosystems, 4352341E) was used as an endogenous control. Target genes were amplified with probes designed by Integrated DNA Technologies (Table S1). Cycle threshold (Ct) values were recorded, normalized to its endogenous control and transformed to relative gene expression value using the 2^−ΔΔCt^ method [50]. Each sample was analysed in triplicate for both target gene and endogenous control. The gene expression levels for each animal was calculated considering the mean from each of these triplicates.

## 3 Methods – human intervention study

### 3.1 Human intervention study outline

This study has a parallel-controlled design. In total, 150 individuals were screened, after which 124 were randomized into the treatment groups (Placebo: *n* = 50; Treatment: *n* = 74). The aim of the first visit of the participant was to assess participants for their eligibility to participate in the study and explain which procedures would be undertaken. Subjects were given an appointment for the next visit within a 3-week period. At the second visit, all baseline data and biologics were recorded, which was also done after 6 (visit 3) and 12 weeks (visit 4) of placebo or *B. longum* APC1472 treatment. Vital signs, anthropometric measurements and medical history were recorded. For women of childbearing age, a urine sample was collected for a pregnancy test. Fasting blood samples (20 mL) were collected to assess glucose, insulin, HbA1c, lipid profiles, satiety/appetite hormone profiles, and inflammatory profiles. Saliva samples were collected for the assessment of the cortisol awakening response, as well as a stool sample for the microbiota analysis and short-chain fatty acid (SCFA) quantification. Questionnaires were administered to assess self-reported perceived stress, anxiety, hunger/satiety, exercise and diet.

Participants were asked to take one capsule per day, providing a daily dose of 1 × 10^10^ CFU. Subjects, study facilitators, nurses and research analysts were kept blind as to in which group they belonged. The randomisation of treatment schedules was carried out by a computer-generated program. The remaining study product was collected to check for compliance following visits 3 and 4 [51].

### 3.2 Inclusion and exclusion criteria

The inclusion criteria were as follows: subjects had to give written informed consent; had to be between 18 and 65 years of age; had a BMI between 28 and 34.9; had a W/H ratio ≥0.88 for males and ≥0.83 for females; had to be willing to consume the investigational product daily for the duration of the study. Subjects were excluded if they were pregnant, lactating, or female and wish to become a parent during the study; regularly took probiotics; were hypersensitive to any of the components of the test product; were severely immune-compromised (i.e. HIV positive, transplant patient, antirejection medications, on a steroid for >30 days, or underwent chemotherapy or radiotherapy within the last year); had Type 1 or Type 2 Diabetes Mellitus; had a history of bariatric surgery; had taken anti-obesity medication in the previous 12-weeks; were actively, or has within the last 3 months, participating in a weight loss program or incurred a weight change of more than 3 kg during the past 3 months; had a life-threatening illness; was on Metformin, anti-psychotic drugs or any medication that the investigator determined could impact the results of the study; had commenced use of anti-hypertensive drugs, anti-depressive drugs, statins or any other medication that the investigator determined could impact the results of the study within 3-months of randomisation date; had a history of co-existing gastrointestinal, and/or gynaecological, and/or urologic pathology (e.g. colon cancer, colitis, Crohn's Disease, celiac, Endometriosis, prostate cancer) or lactose intolerance; had a history of drug and/or alcohol abuse; was currently, or planning, to participate in another study during the study period; had a history of non-compliance; had been on antibiotics in the 12-weeks prior to randomisation; or consumed vitamin D supplements (>5000 IU/d). 17.3% of all screened participants were excluded due to these exclusion criteria.

Subjects were removed from the study if they independently elected to withdraw; he/she developed any condition which contravened the original criteria; or was considered at any point to be unsuitable to continue the study, at the discretion of the investigator.

### 3.3 Study setting and ethical approval

**Fig. 1:**
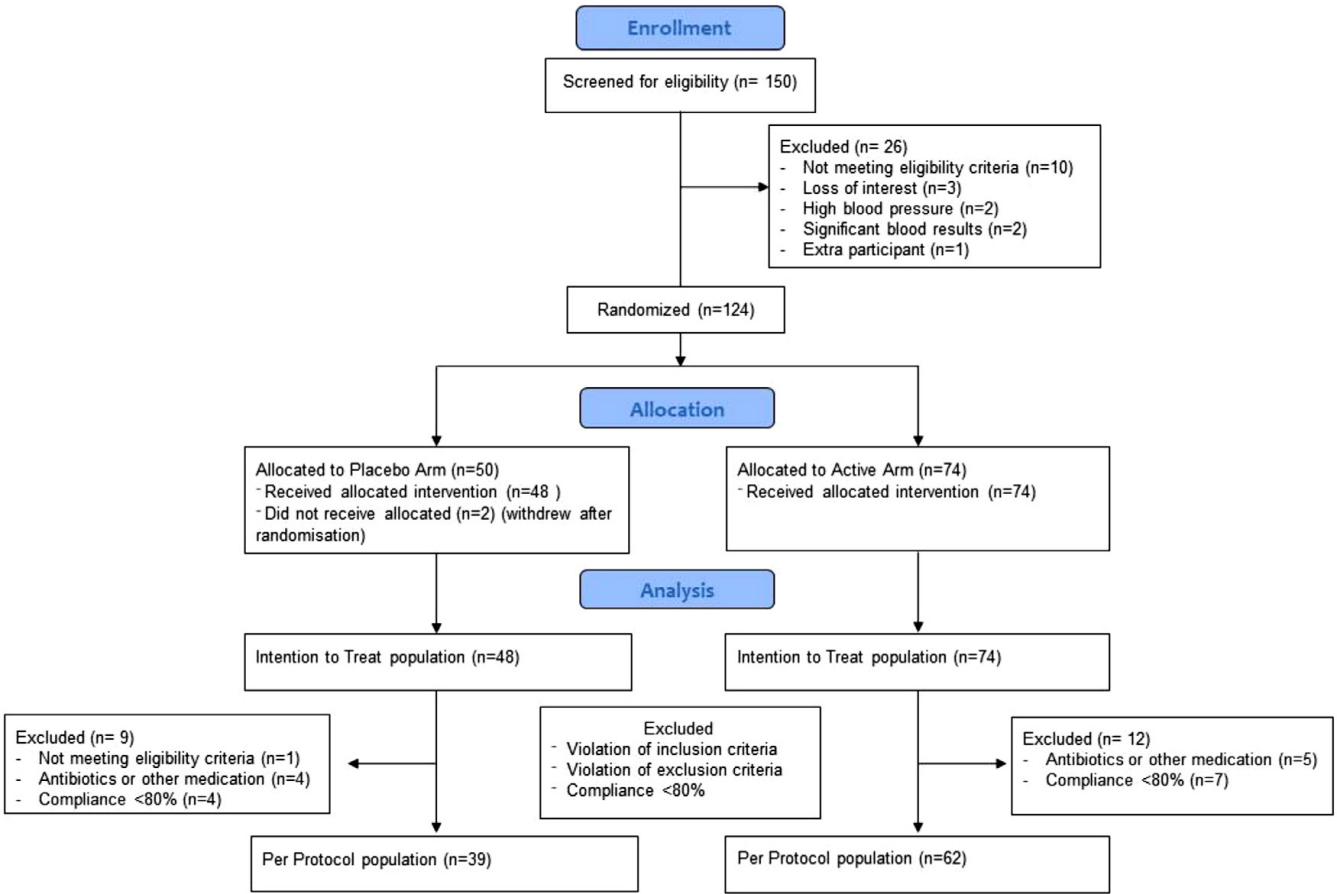
Consort diagram. Number of healthy overweight/obese participants that were assessed for eligibility and excluded or allocated to the trial, treated, followed, and analysed.

The study was conducted in accordance with the ethical principles set forth in the current version of the Declaration of Helsinki (seventh version, October 2013), the International Conference on Harmonization E6 (R2) Good Clinical Practice (ICH GCP, November 2016) and all applicable local regulatory requirements (i.e. Clinical Research Ethics Committee of the Cork Teaching Hospitals). This study was registered with ClinicalTrials.gov (NCT04042181). The CONSORT diagram of this study is depicted in Fig. 1, the study layout is depicted in **Figure S2**. This study was run by Atlantia Food Clinical Trials (Cork, Ireland) (study reference: AFRCO-088).

### 3.4 Randomisation and blinding

The investigational product arrived on site labelled with randomisation number. A randomisation list was generated by an independent statistician. Participants were assigned a randomisation number in chronological order from this randomisation list. The study team, participants and researchers were unaware which randomisation numbers were active or placebo. Blinding was undone after all data had been analysed.

### 3.5 Study recruitment

Subjects were recruited through the database of Atlantia Food Clinical Research Trials, general practitioners’ offices and by posting adverts in local newspapers. Subjects underwent an initial phone screen. Eligible subjects were scheduled for a screening visit. Subjects received €300 upon completion of the study to cover costs and expenses incurred.

### 3.6 Product formulation and dosage

*Bifidobacterium longum* has been granted Qualified Presumption of Safety (QPS) status by the European Food Safety Authority (EFSA). *B. longum* APC1472 grown culture and the corresponding placebo were freeze-dried (Sacco SRL, Italy) and provided as hydroxypropylmethylcellulose (HPMC) capsules in PE bottles (Nutrilinea, Italy). The freeze-dried powder of the strain was blended with standard food-grade excipients to achieve the target dose of 1 × 10^10^ CFU, which was based on previous publications [52], [53], [54]. The excipients consisted of corn starch, magnesium stearate and silicon dioxide. The probiotic formulation consisted of *B. longum* APC1472, whereas the placebo contained maltodextrin. The product was stored at −20 °C until distributed to the study participant and the participant was instructed to keep the product refrigerated. Participants returned any leftover product at their next visit, and the excess product was counted to check for compliance.

### 3.7 Collection and analysis of blood samples

Fasting blood samples were taken into EDTA tubes, fasting defined as refraining from food overnight (at least 10 h), however drinking water was allowed throughout the duration of the fast. Samples for the analysis of active ghrelin were immediately treated with AEBSF (final concentration 1 mg/mL, Sigma, A8456), centrifuged and the resulting plasma was treated with HCl (final concentration 0.05 N). Blood samples for the analysis using the U-PLEX assays were treated with DPP-IV inhibitor (final concentration 1%, Sigma, DPP4) and centrifuged. Blood plasma samples for other analyses did not undergo any additional processing, except for centrifugation. Centrifugation was performed at 1000 g for 10 min at 4 °C, after which samples were aliquoted and either processed or stored at −80 °C for future analysis.

Blood plasma from visit 1 was used to measure urea, creatinine, bilirubin, alanine aminotransferase, alkaline phosphatase, aspartate aminotransferase, gamma-glutamyl transferase, total protein, albumin, globulin, calcium, magnesium, phosphate, uric acid, cholesterol, HDL cholesterol, LDL cholesterol, total triglycerides, glucose, full blood count + 5-part diff. Safety blood, haematology and biochemistry parameters were analysed by Biomnis-Eurofins Ireland.

Blood from visits 2, 3 and 4 was used to measure total cholesterol, LDL, HDL, triglycerides HbA1c, glucose and insulin by Biomnis-Eurofins Ireland. Furthermore, blood plasma was assessed for active ghrelin levels using an ELISA (EMD Millipore, EZGRA-88BK) which was performed according to the manufacturer's instructions. Plates were read at 405 nm with a correction at 590 nm using the synergy HT plate reader (Biotek instruments). Blood plasma was also assessed for metabolic and inflammatory biomarkers using custom U-PLEX assays (MSD, K151ACM-2), which were also performed according to the manufacturer's instructions. Blood plasma samples were diluted 1:3 for the U-PLEX assays. U-PLEX markers were linked as following; Plate 1: 1) Leptin, 2) PYY, 3) GLP-1 – total, 4) IFNγ, 5) Il-4, 7) TNF-α, 8) Il-10, 9) C-peptide, 10) Ghrelin – total; Plate 2: 1) GLP-1 – active. The working solution was supplemented with DPP-IV inhibitor (final concentration 1%, Sigma, DPP4). Plates were read using the MESO QuickPlex SQ 120. Duplicates with ≥ 20% coefficient of variability were re-analysed. Samples did not undergo any additional freeze-thaw cycles.

### 3.8 Collection and analysis of cortisol awakening response samples

To monitor the cortisol awakening response, saliva from visits 2 and 4 was collected in Salivette devices (Sarstedt, 51.1534.500) immediately upon awakening, and after 30, 45 and 60 min. Participants were instructed to keep samples in the fridge until delivery at the visit time, after which they were centrifuged at 1500 g for 5 min, the saliva was harvested and immediately stored at −80 °C. Salivary cortisol concentrations were quantified using ELISA kits (Enzo life sciences, ADI-901–071), which were performed according to the manufacturer's instructions. Saliva samples were diluted 1:2. Plates were read at 405 nm with a correction at 580 nm using the synergy HT plate reader (Biotek instruments). Duplicates with ≥ 20% coefficient of variability were re-analysed. Samples did not undergo any additional freeze-thaw cycles. Cortisol awakening response was calculated using the area under the curve increase (AUCi). Briefly, data from the 30-, 45- and 60-minute time-points were normalized (delta) to the samples taken immediately upon awakening, after which the sum was taken of the 30-, 45- and 60-minute time-points [52].

## 4 Methods – murine and human microbiota

### 4.1 Murine and human microbiota sequencing

Murine caecal DNA was isolated using the QIAamp Fast DNA Stool Mini kit (Qiagen) as previously described and kept at −20 °C until further analysis [53]. Isolated DNA was quantified on a NanoDrop ND2000 spectrophotometer (Thermo Scientific, DE) and used for 16S ribosomal RNA sequencing by Illumina MiSeq System (Illumina Inc., USA) according to the manufacturer's instructions. Briefly, PCR amplicons (primers for V3-V4 hypervariable region of the 16S rRNA gene: F (5′-TCGTCGGCAGCGTCAGATGTGTATAAGAGAC AGCCTACGGGNGGCWGCAG-3′) and R (5′-GTCTCGTGGGCTCGGAGATGTGTATA AGAGACAGGACTACH VGGGTATCTAATCC-3′) were purified and libraries prepared as previously described [53]. Briefly, the 16S V3-V4 amplicons were generated using Kapa HiFi HS ready mix and purified using the Agencourt AMPure XP system (Beckman Coulter Genomics, Takeley, UK). The Nextera XT Index Kit (Illumina Inc., USA) was used to barcode each sample. PCR products were cleaned using AMPure XP beads and a magnetic 96-well plate. Final barcoded amplicons were measured using the Qubit dsDNA High Sensitivity assay kit on the Qubit 3.0 fluorometer, diluted to 5 ng/µL and pooled. The PCR products from both PCR steps (Amplicon & Indexing) were visualised in agarose gels stained with SYBR Safe DNA gel stain (Invitrogen). Samples were sequenced at Clinical-Microbiomics, Denmark on the Illumina MiSeq platform using a 2 × 300 bp kit. After sequencing, reads were assembled, processed and analysed as previously described [53]. In the microbiota composition analysis, LDA Effect Size (LEfSe: Linear Discriminant Analysis Effect Size) was used as an algorithm with default settings on the interface Galaxy (http://huttenhower.sph.harvard.edu/lefse/) [54] to identify taxa with differentiating abundances. The differentially abundant features are ranked by effect size after undergoing linear discriminant analysis (LDA), using an effect size threshold of 2 (log10 scale). In non-technical terms, LEfSe pre-selects features that are different between groups and then tries to fit a model to see how well these features explain the groups. The score is an average between the effect size and how well the model fits, after which they are transformed to a value between −6 and 6. Principal coordinates Analysis (PCoA) was performed based on Bray-Curtis beta diversity distances using the Adonis function in the “vegan” (2.4–3) package for R (version 3.3.1).

For the human intervention study, faecal sample collection and DNA extraction was performed as previously described (see supplementary material for details) [55]. The DNA samples were processed according to the Illumina 16S Metagenomic Sequencing Library Preparation instructions as described above for the murine DNA samples. Final barcoded amplicons were measured using the Qubit dsDNA High Sensitivity assay kit on the Qubit 3.0 fluorometer, diluted to 8.3 ng/µL, pooled and sent for sequencing. Microbiome analysis was carried out in R (version 3.6.1) with Rstudio (version 1.2.1335). DADA2 was used to denoise and call amplicon sequence variants (ASVs). Taxonomy was assigned using the SILVA SSUREf database version 132. ASVs unknown on a genus level were excluded, as well as ASVs present in two or fewer samples. The ALDEx2 library used to compute the centred log-ratio transformed values of the remaining taxa [56]. For principal components analysis (PCA), a pairwise implementation of the adonis() PERMANOVA function in the vegan library followed by the Bonferroni-Holm correction was used to test for difference in β-diversity in terms of Aitchison distance (source: Oksanen, Jari, et al. "Package ‘vegan’." Community ecology package, version 2.9 (2013): 1–295). Differential abundance was assessed using a pairwise implementation of the aldex.test() function, followed by Benjamini-Hochberg correction. In all cases, a q-value < 0.1 was considered significant. α-diversity was computed using the iNEXT library [57].

### 4.2 Faecal SCFA quantification

Faecal samples were homogenised with acidified water (HCl pH 3) at a ratio of 1:7.5 w/v and analysed by gas chromatography flame ionisation detection (GC-FID) using a Varian 3800 GC system, fitted with an Agilent DB-FFAP column (30 mL x 0.32 mm ID x 0.25 μm df; Agilent) and a flame ionisation detector with a CP-8400 auto-sampler. Helium was employed as the carrier gas at an initial flow rate of 1.3 mL/min. The initial oven temperature was 50 °C, was maintained for 30 s, raised to 140 °C at 10 °C/min and held for 30 s, before being increased to 240 °C at 20 °C/min, and held for 5 min (total run time 20 min). The temperatures of the detector and the injection port were 300 °C and 240 °C, respectively. A split-less injection of 0.2 µL was carried out for each sample or standard using a 10 µL syringe (Agilent) installed to a CP-8400 auto-sampler (Varian). A 5 m guard column was installed between the injector and analytical column (Restek). Peak integration was performed using Varian Star Chromatography Workstation version 6.0 software. Vials containing 1800 µL of water were run between each sample duplicates as blanks to control for any potential carryover. Standards were included in each run to maintain the calibration. For further details on sample and standards preparation see supplementary information.

### 4.3 Questionnaires

Using self-report scales, participants were assessed for perceived stress using Cohen's Perceived Stress Scale and anxiety and depression using the Hospital Anxiety and Depression Scale (HADS) at baseline, after 6 and after 12 weeks, as previously described[58], [59]. In addition, satiety/hunger was determined using a visual analogue Hunger/Satiety scale, physical activity using the International Physical Activity Questionnaire (IPAQ) [60]. Nutrient intake was assessed using a Food Frequency Questionnaire (FFQ), as previously described [61].

### 4.4 Statistical analysis

Preclinical data were assessed for normality using the Shapiro-Wilk test. Normally distributed data were analysed using a two-way ANOVA, followed by Fisher's least significant difference (LSD) post hoc test. Non-parametric datasets were analysed using the Kruskal-Wallis test, followed by the Mann-Whitney U test with Bonferroni adjustment of p-values. Body weight changes and glucose levels in glucose tolerance test were analysed with a two way repeated-measures ANOVA (with Diet and Probiotic as two independent factors and Time as a repeated-measured factor), followed by LSD post hoc test at each time point. Statistical analysis was performed using SPSS software (IBM SPSS statistics 22). Preclinical data are represented as mean ± SEM.

For the human intervention study, differences between the treatment and placebo groups at the last visit (i.e. visit 4) were analysed using an analysis of covariance (ANCOVA), correcting for baseline variance (i.e. visit 2) and sex. Comparisons between baseline measurements (visit 2) and post-intervention measurements (visit 4) were analysed using an unpaired student's T-test. Analyses were performed on the intention to treat populations. Statistical analysis was performed using SPSS software version 26 (IBM Corp). Data in table are presented as mean ± SEM or 95% CI. P-Values <0.05 were considered statistically significant. For significant associations, a Benjamini-Hochberg procedure was performed with a threshold of *q* <0.1. Partial eta-squared (η^2^) was used to estimate effect size [62]. Effect sizes were interpreted as following: η^2^ ≤ 0.06 was considered small, 0.06 > η^2^ ≤ 0.14 was considered moderate, η^2^ ≥ 0.14 was considered large.

## 5 Results

### 5.1 *B. longum* APC1472 decreases body weight gain and fat depots accumulation in obese mice

**Fig. 2:**
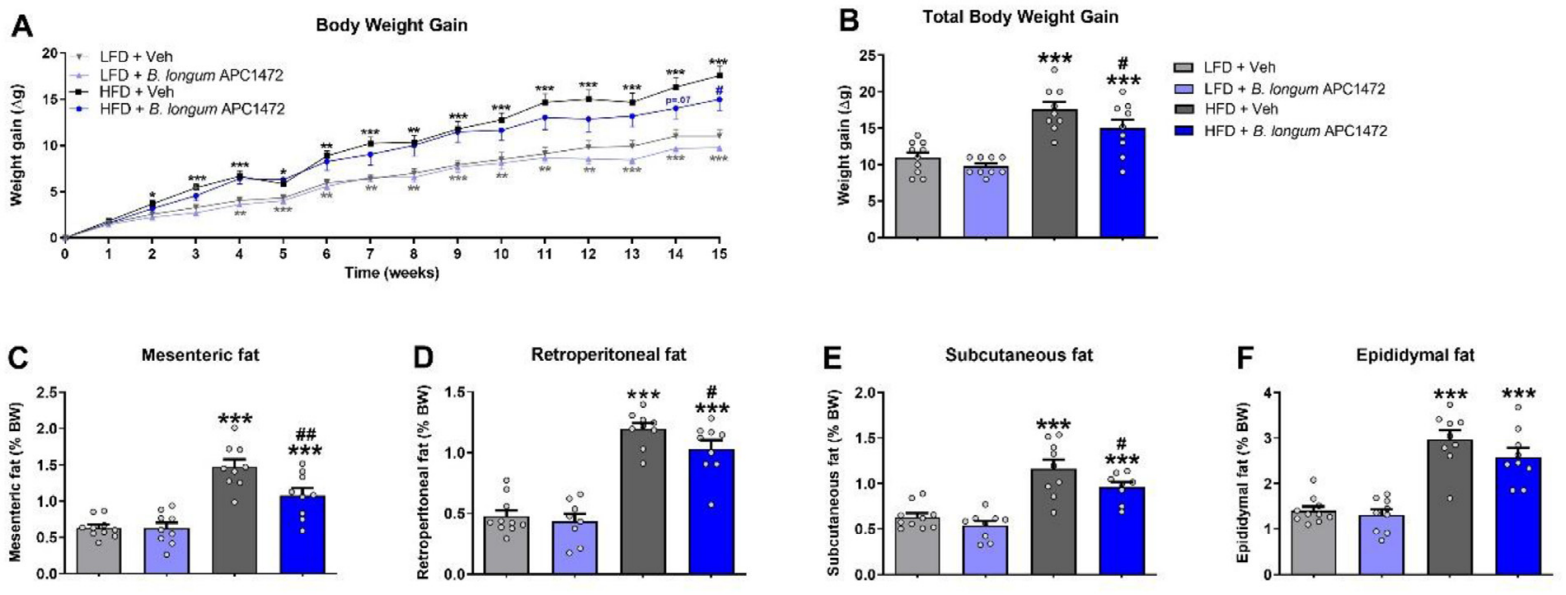
Effects of Bifidobacterium longum APC1472 on body weight and fat depots accumulation in mice. (A) Weekly body weight gain, (B) total body weight gain and (C) mesenteric, (D) retroperitoneal, (E) subcutaneous and (F) epididymal fat depots accumulation (% of total body weight) in control mice treated with drinking water containing sterile PBS (2% v/v) and glycerol (0.5% v/v) and fed a control low-fat diet (LFD) (*n* = 10) or a high-fat diet (HFD) (*n* = 9) and in mice treated with B. longum APC1472 in drinking water (2 × 10^8^ CFU/mL) and fed a LFD (*n* = 9 in A, B, C, E and F; *n* = 8 in D) or a HFD (*n* = 9 in A, B, C, D, and F; *n* = 8 in E) for 15 (A, and B) or 16 weeks (C, D, E and F). Data are shown as mean ± SEM.. Data are significant different (*p*<0.05) accordingly to Repeated Measures ANOVA (A) or two-way ANOVA followed by LSD post-hoc test (B, C, D, E and F). * indicates significant diet treatment effect (*p<0.05, **p<0.01, ***p<0.001) and ^#^ indicates significant B. longum APC1472 treatment effect (^#^*p*<0.05, ^##^*p*<0.01).

*B. longum* APC1472 decreased body weight gain after 15 weeks of administration (*F* (1, 33) = 4.751, *p* = 0.037) (Fig. 2**A,** 2**B**). HFD feeding increased caloric intake (F (1, 15) = 9.229, *p* = 0.008) (**Figure S1C**), body weight (F (1, 33) = 29.715, *p* < 0.001) (Fig. 2**A**) and fat depot accumulation (mesenteric (F (1, 33) = 61.328, *p* < 0.001), retroperitoneal (F (1, 32) = 128.409, *p* < 0.001), subcutaneous (*F* (1, 31) = 124.091, *p* < 0.001) and epididymal (*F* (1, 33) = 81.673, *p* < 0.001)) (Fig. 2**C, D, E, F**). Pairwise comparisons showed a significant decreased body weight effect of *B. longum* APC1472 in HFD-fed mice (*p =* 0.047) (Fig. 2**B**), which was independent of caloric intake (**Figure S1**). Furthermore, the administration of *B. longum* APC1472 significantly reduced fat depot accumulation (mesenteric (*F* (1, 33) = 5.908, *p* = 0.021), and subcutaneous (*F* (1, 33) = 4.270, *p* = 0.047)) (Fig. 2**C, D, E, F**). Finally, pairwaise comparisons revealed a significant decreased fat depot accumation effect of *B. longum* APC1472 administration in HFD-fed mice (mesenteric *p* = 0.002, retroperitoneal *p* = 0.05 and subcutaneous *p* = 0.023).

### 5.2 *B. longum* APC1472 administration improves glucose tolerance, circulating levels of leptin and corticosterone in obese mice

**Fig. 3:**
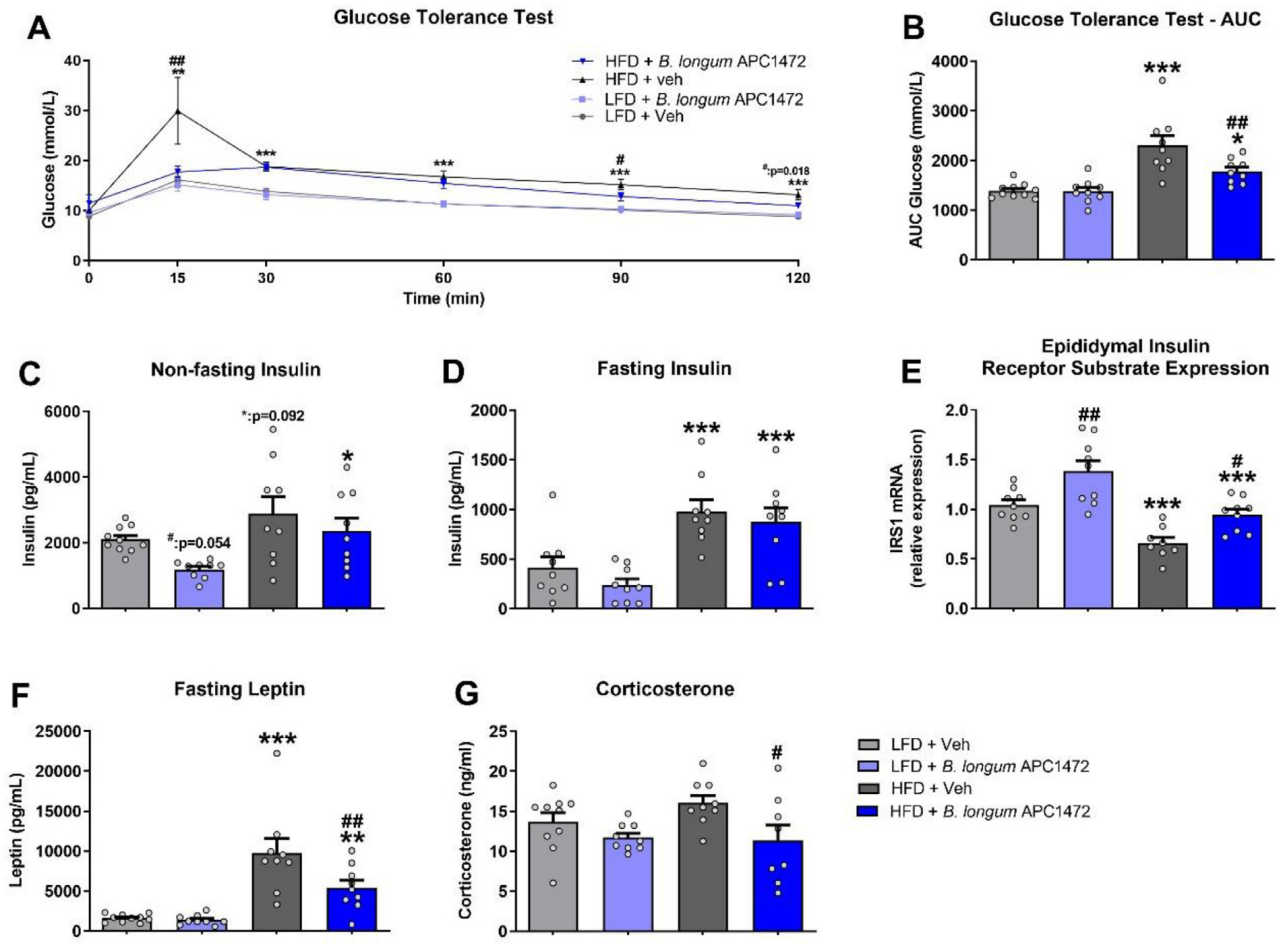
*Bifidobacterium longum* APC1472 improved glucose tolerance, leptin plasma levels and stress-induced corticosterone circulating levels in high-fat diet-induced obesity in mice. (A and B) Glucose tolerance test (GTT) glucose curve and area under the curve (AUC) after 1 g/kg glucose challenge, (C and D) non-fasting and fasting insulin plasma levels, (E) fasting leptin plasma levels, (F) epididymal fat insulin receptor substrate (IRS)−1 mRNA expression and (G) fasting-induced corticosterone plasma in control mice treated with drinking water containing sterile PBS (2% v/v) and glycerol (0.5% v/v) and fed a control low-fat diet (LFD) (*n* = 10 in A, B, C, E, F and G) or a high-fat diet (HFD) (*n* = 9 in A, B, C, D, E and G; *n* = 8 in F) and in mice treated with *B. longum* APC1472 in drinking water (2 × 10^8^ CFU/mL) and fed a LFD (*n* = 9 in A, B, C, D, E and F; *n* = 8 in G) or a HFD (*n* = 9 in A, B, C, D, E and F; *n* = 8 in G) for 15 (A, B,C) or 16 weeks (D, E, F and G). Data are shown as mean ± SEM. Data are significant different (*p*<0.05) accordingly to Repeated Measures ANOVA (A) or two-way ANOVA followed by LSD post-hoc test (B, C, D, E, F and G). * indicates significant diet treatment effect (*p<0.05, **p<0.01, ***p<0.001) and ^#^ indicates significant *B. longum* APC1472 treatment effect (^#^*p*<0.05, ^##^*p*<0.01).

Effects of HFD feeding (*F* (5, 155) = 3.321, *p* = 0.007) and *B. longum* APC1472 supplementation (*F* (5, 155) = 4.792, *p* < 0.001) were observed, as well as an interaction effect between these two factors and time (*F* (5, 155) = 3.307, *p* = 0.007) in the glucose tolerance test. Supplementation with *B. longum* APC1472 normalized glucose levels after 15 mins of glucose administration in HFD-fed obese mice (*p* = 0.006) and significantly decreased glucose after 90 (*p* = 0.019) and 120 min (*p* = 0.018) respectively (Fig. 3**A**) as determined by 2 way ANOVAs at each individual timepoint. Moreover, HFD feeding (*F* (1, 33) = 29.761, *p* < 0.001), *B. longum* APC1472 (*F* (1, 33) = 4.425, *p* = 0.043) and interaction effects between these two factors (*F* (1, 33) = 5.337, *p* = 0.027) were also observed when analysing the area under the curve (AUC) for glucose levels (Fig. 3**B**), with *B. longum* APC1472 administration significantly reducing AUC in HFD-fed mice (*p* = 0.003) as determined by post-hoc comparison (Fig. 3**B**). In addition, both a HFD feeding (F (1, 33) = 9.167, *p* = 0.005) and a *B. longum* APC1472 effect (*F* (1, 33) = 4.796, *p* = 0.036) were observed for non-fasting insulin levels (Fig. 3**C)**. Interestingly, *B. longum* APC1472 reduced non-fasting insulin levels in LFD-fed mice (*p* = 0.054) but not in HFD-fed mice (Fig. 3**C)**. However, for fasting glucose levels, only a HFD feeding effect was observed (F (1, 32) = 29.153, *p* < 0.001) (Fig. 3**D**). Moreover, both a HFD feeding (F (1, 31) = 30.926, *p* < 0.001) and a *B. longum* APC1472 effect (*F* (1, 31) = 17.917, *p* < 0.001) were observed for epididymal insulin receptor substrate 1 (*IRS-1*) expression (Fig. 3**E**). Post-hoc comparisons determined that *B. longum* APC1472 significantly reduced *IRS*-1 expression in both LFD (*p* = 0.002) and HFD-fed mice (*p* = 0.011) (Fig. 3**E**). Both a HFD feeding (*F* (1, 33) = 38.023, *p* < 0.001) and a *B. longum* APC1472 (*F* (1, 33) = 5.340, *p* = 0.027) effect as well as an interaction effect (F (1, 33) = 4.237, *p* = 0.048) were observed for fasting leptin levels (Fig. 3**F)**. The effect of HFD on leptin levels was attenuated by *B. longum* APC1472 treatment (*p* = 0.004). Finally, we found a significant *B. longum* APC1472 treatment effect (*F* (1, 32) = 7.774, *p* = 0.009) for plasma corticosterone levels (Fig. 3**G**). Administration of *B. longum* APC1472 significantly decreased plasma corticosterone levels in HFD-fed mice (*p* = 0.011) (Fig. 3**G**), which may have contributed to its overall impact on glucose homeostasis [98].

### 5.3 *B. longum* APC1472 induces changes of hypothalamic neuropeptide expression in mice

Analysis of the gene expression levels of hypothalamic neuropeptides involved in appetite modulation revealed a significant HFD effect on the gene expression of the orexigenic peptide agouti-related protein (*AgRP*) (*F* (1, 33) = 10.412, *p* = 0.003) but a non-significant reduction in neuropeptide Y (*NPY*) expression (**Figure S3**). Interestingly, both a *B. longum* APC1472 effect (*F* (1, 33) = 7.820, *p* = 0.009) and an interaction effect (*F* (1, 33) = 5.881, *p* = 0.021) were observed for cocaine- and amphetamine-regulated transcript (*CART*) expression (**Figure S3**). Indeed, *B. longum* APC1472 administration significantly reduced *CART* expression in HFD-fed mice (*p* = 0.001). While a reduced expression was observed for the anorexigenic pro-opiomelanocortin (*POMC*) gene expression in HFD-fed animals treated with *B. longum* APC1472 compared to HFD-fed, this did not reach statistical significance. Finally, no significant change in leptin (*LEP-R*) nor ghrelin (*GHS-R1a*) receptor expression was observed (**Figure S3**).

### 5.4 Human intervention study population

In the human study, no significant differences were observed in weight, BMI, W/H ratio, age, height, sex, ethnicity, mode of delivery, alcohol consumption, and medical/surgical history at baseline between *B. longum* APC1472 treatment and placebo groups, as well as compliance (Table 1). We did observe an increased prevalence of concomitant medical or nutritional supplement consumption in the treatment group (48.6%) compared to the placebo group (33.3%). In addition, we also observed differences in the socioeconomic profile where there was a lower prevalence of employers and managers in the treatment group (2/74) compared to the placebo group (4/48). Similarly, we observed a lower prevalence of past smokers in the treatment group (28/74) compared to the placebo group (9/48). In conclusion, the baseline characteristics of our placebo group and *B. longum* APC1472 group are mostly the same.

**Table 1:** Baseline characteristics of subjects in the placebo and treatment arms at visit 1 (screening visit).

Variable	Placebo (*n* = 48, mean ± STD)	*B. longum* APC1472 (*n* = 74, mean ± STD)
Weight (kg)	87.9 ± 1.7	89.0 ± 1.3
BMI	31.2 ± 0.3	30.8 ± 0.2
W/H ratio	0.95 ± 0.01	0.96 ± 0.01
Age (years)	46.3 ± 9.9	44.9 ± 11.4
Height (m)	1.67 ± 0.10	1.70 ± 0.09
Sex (no. of subject (%))		
Male	19 (39.6%)	34 (45.9%)
Female	29 (60.4%)	40 (54.1%)
Race or ethnicity (no. of subject (%))		
Caucasian	48 (100%)	73 (98.6%)
Arabic	0 (0%)	1 (1.4%)
Socioeconomic status (no. of subject (%))		
Non-manual	15 (31.3%)	21 (28.4%)
Lower Professional	14 (29.2%)	19 (25.7%)
Manual skilled	4 (8.3%)	8 (10.8%)
Semi-skilled	4 (8.3%)	8 (10.8%)
Employers and managers	4 (8.3%)	2 (2.7%)
Own account workers	3 (6.3%)	7 (9.5%)
Higher Professional	3 (6.3%)	5 (6.8%)
All others gainfully occupied and unknown	1 (2.1%)	2 (2.7%)
Farmer	0 (0%)	1 (1.4%)
Unskilled	0 (0%)	1 (1.4%)
Smoking status (no. of subject (%))		
Non-smoker	22 (45.8%)	40 (54.1%)
Past smoker	17 (35.4%)	28 (37.8%)
Current smoker	9 (18.8%)	6 (8.1%)
Alcohol consumption (mean ± SEM)		
Units per week	4.97 ± 0.68	4.31 ± 0.46
Currently on concomitant medical or nutritional supplements (no. of subject (%))		
Yes	16 (33.3%)	36 (48.6%)
No	32 (66.7%)	38 (51.4%)
Compliance (% product consumed)		
Week 6	95.8 ± 1.2	97.9 ± 0.8
Week 12	94.0 ± 2.0	97.2 ± 1.2

Abbreviations: BMI = Body-mass index; W/H ratio = waist-to-hip ratio.

Physical activity and food intake patterns were also assessed throughout the study using self-report questionnaires (**Table S2, S3**). No differences in physical activity levels or calorie, macro- and micronutrient intake were observed over the 12-week treatment period or between the placebo and *B. longum* APC1472 group.

### 5.5 Adverse events

There were seven adverse events (6 placebo participants and 1 treatment participant) that were possibly related to the investigational product. The adverse event of the treatment participant was constipation. The remaining 6 adverse events for placebo participants were; gastrointestinal discomfort and increased appetite; bloating; increased flatulence; aches in joints and increased temperature; rash on knees, elbows, scalp and red blotches on chest & upper arm.

### 5.6 *B. longum* APC1472 does not affect BMI and W/H ratio in humans

**Fig. 4:**
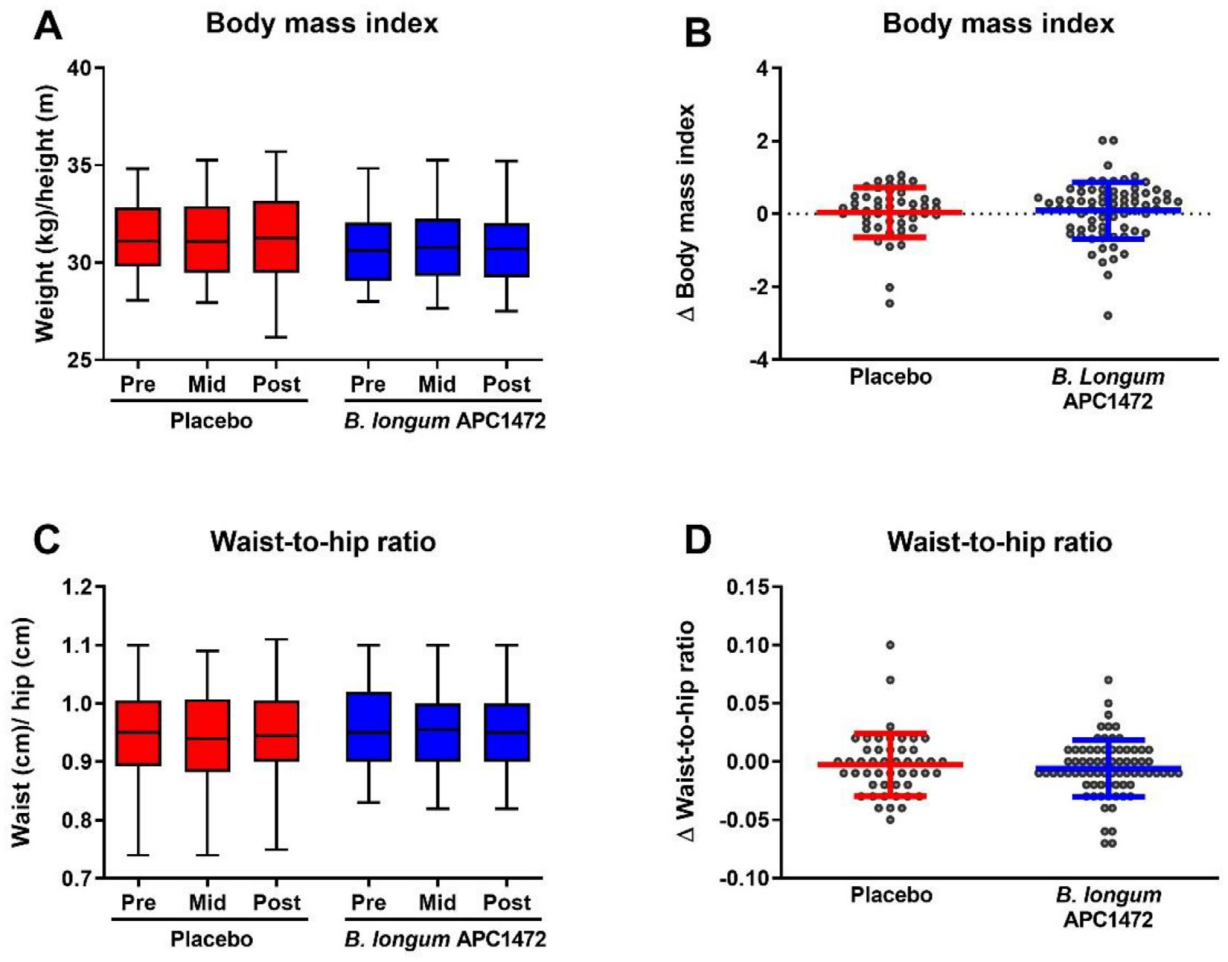
*B. longum* APC1472 supplementation does not impact BMI and W/H ratio in overweight and obese individuals. Body mass index (BMI) (A, B) and waist-to-hip ratio (W/H ratio) (C, D) were measured as the beginning of the study (pre), after 6 weeks (mid) and after 12 weeks (post) of treatment. All BMI and W/H ratio data are depicted of all 3 timepoints (A, C), as well as the change after 12 weeks compared to at the beginning of the study (B, D). Data are depicted as boxplot or scatter dot plot, where the dots depict individual datapoints, with *n* = 48 for the placebo group and *n* = 74 for the *B. longum* APC1472 treatment group.

The primary outcome of this study was to investigate whether *B. longum* APC1472 supplementation could alter BMI, and a secondary outcome of change in W/H ratio was included to support the primary outcome. However, no differences were observed in BMI and W/H ratio over the 12-week treatment period, or between the placebo and *B. longum* APC1472 treatment groups (Fig. 4).

### 5.7 *B. longum* APC1472 improves fasting glucose levels independent of other blood markers of energy metabolism and satiety in humans

**Fig. 5:**
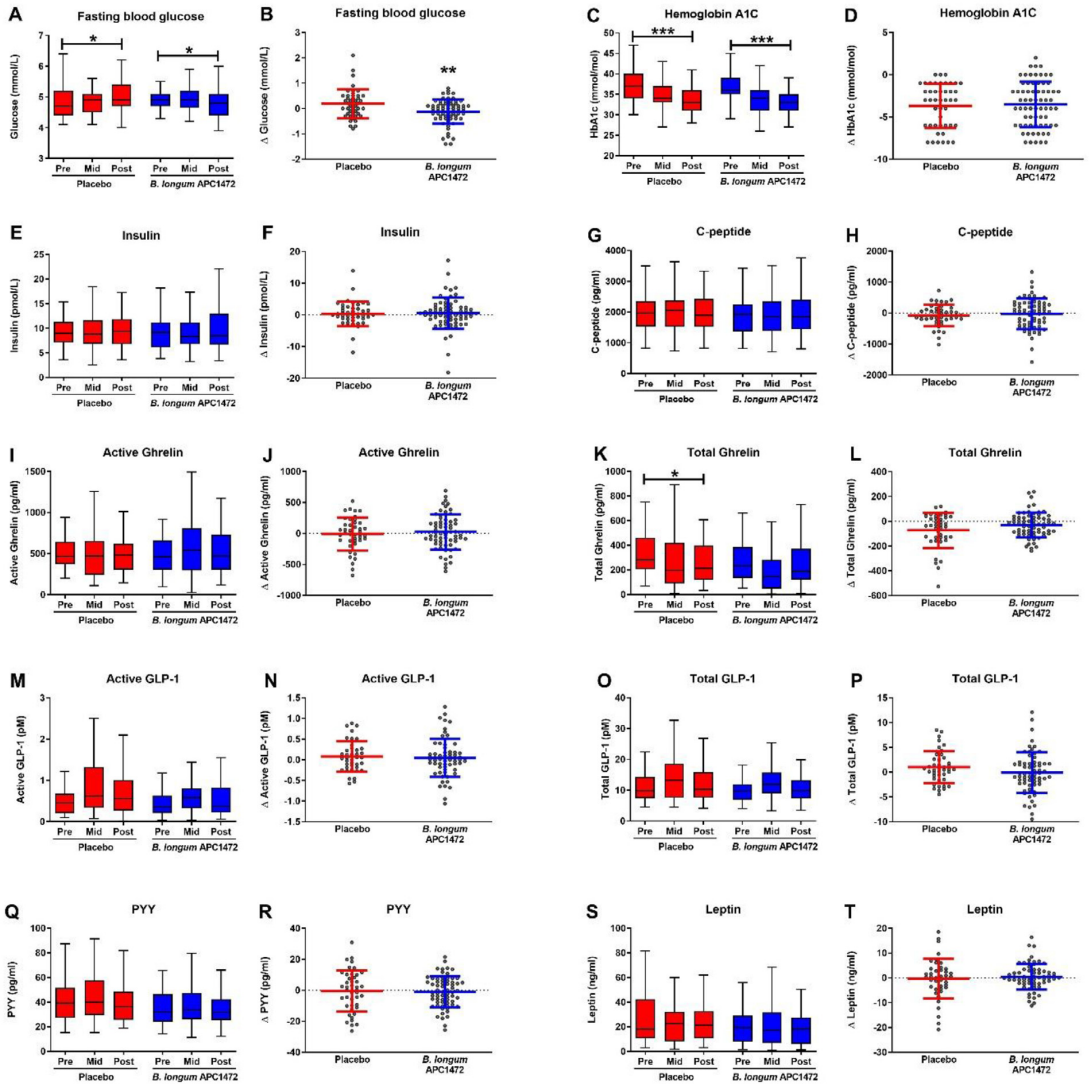
*B. longum* APC1472 supplementation reduces fasting blood glucose levels in overweight and obese individuals. Markers associated with host metabolism and satiety were measured as the beginning of the study (pre), after 6 weeks (mid) and after 12 weeks (post) of treatment. All data are depicted of all 3 timepoints (A, C, E, G, I, K, M, O, Q, R), as well as the change after 12 weeks compared to at the beginning of the study (B, D, F, H, J, L, N, P, R, T). Data are depicted as boxplot or scatter dot plot, where the dots depict individual datapoints, with *n*= 48 for the placebo group and *n* = 74 for the *B. longum* APC1472 treatment group. * indicates a significant effect (*p<0.05, **p<0.01, ***p<0.001).

We subsequently measured markers associated with host energy metabolism and satiety as part of the secondary and exploratory outcome measures (Fig. 5 and **Table S4** for full statistical results). Here we observed that both the *B. longum* APC1472 and the placebo arm reduced fasting glucose levels over the 12-week treatment period (Fig. 5**A**). However, glucose levels were 0.266 mmol/L (95% CI [−0.44, −0.09]) lower in the *B. longum* APC1472 group compared with the placebo group (*F*(1112) = 9.073, *p* = 0.003; *q* = 0.075) (Fig. 5**B**). The effect size of the *B. longum* APC1472-induced decrease was moderate (η^2^ = 0.075). We also observed that HbA1c levels decreased over the 12-week treatment period in both the placebo group (*t*(62.372) = 4.277, *p* < 0.001) and *B. longum* APC1472 treatment group (*t*(85.983) = 5.787, *p* < 0.001) (Fig. 5**C**). However, there were no differences between the groups, indicating that the decrease in HbA1c levels is most likely explained by the 12-week treatment period or placebo effect. No changes were observed in other biomarkers of host energy metabolism such as insulin, C-peptide, ghrelin (active and total), GLP-1 (active and total), PYY and leptin levels (Fig. 5**E-T**).

### 5.8 *B. longum* APC1472 does not influence human lipid and inflammatory profiles in humans

It is well-known that obesity is associated with metabolic syndrome, hypertension and hyperlipidaemia [63]. *B. longum* APC1472 did not impact lipid profiles (i.e. cholesterol, triglycerides and LDL), and inflammatory profiles (i.e. IL-10, TNF-α and IFNγ) compared to the placebo group (Table 2). In addition, vital signs remained unaltered throughout the study (**Table S5**). These results reveal that *B. longum* APC1472 did not evoke any negative effects on vital signs or induced any inflammation. Interestingly, even though no significant changes were observed in HDL levels over the 12-week treatment period, a small increase in HDL levels was observed in the *B. longum* APC1472 group (*F*(1117) = 3.260, *p* = 0.074). The effect-size of the increase in HDL levels was small (η^2^ = 0.027).

**Table 2:** Human lipid, and inflammatory profiles.

	Placebo (*n* = 48)				*B. longum* APC1472 (*n* = 74)				Placebo vs *B. Longum* APC1472		
Variable	Week 0	Week 6	Week 12	P-value Week 0–12	Week 0	Week 6	Week 12	P-value Week 0–12	Difference (95% CI)	P-value	η2
**Lipid profile**											
**Cholesterol** **(mmol/L)**	5.50 ± 0.12	5.42 ± 0.13	5.34 ± 0.15	0.310	5.45 ± 0.12	5.48 ± 0.12	5.41 ± 0.10	0.691	0.094 (−0.22 to 0.41)	0.556	0.003
**Triglycerides** **(mmol/L)**	1.50 ± 0.13	1.47 ± 0.11	1.41 ± 0.09	0.412	1.46 ± 0.10	1.43 ± 0.08	1.49 ± 0.10	0.778	0.089 (−0.14 to 0.31)	0.432	0.005
**LDL (mmol/L**	3.72 ± 0.13	3.70 ± 0.12	3.75 ± 0.15	0.896	3.73 ± 0.12	3.81 ± 0.11	3.66 ± 0.10	0.473	−0.083 (−0.38 to 0.21)	0.579	0.003
**HDL (mmol/L)**	1.33 ± 0.04	1.34 ± 0.05	1.29 ± 0.05	0.537	1.29 ± 0.04	1.32 ± 0.04	1.36 ± 0.04	0.039	0.091 (−0.01 to 0.19)	0.074	0.027
**Inflammatory profile**											
**IL-10 (pg/ml)**	0.43 ± 0.06	0.46 ± 0.06	0.42 ± 0.05	0.696	0.36 ± 0.03	0.38 ± 0.03	0.38 ± 0.05	0.807	−0.012 (−0.15 to 0.13)	0.864	<0.001
**TNF-α (pg/ml)**	1.14 ± 0.10	1.18 ± 0.12	1.04 ± 0.09	0.236	0.90 ± 0.06	0.88 ± 0.06	0.86 ± 0.06	0.488	−0.013 (−0.14 to 0.11)	0.838	<0.001
**IFNγ (pg/ml)**	9.24 ± 1.38	9.21 ± 1.11	9.6 ± 1.6	0.819	6.80 ± 0.78	5.76 ± 0.50	9.4 ± 3.0	0.383	−0.017 (8.29 to 8.26)	0.997	<0.001

### 5.9 *B. longum* APC1472 does not affect satiety, mood, perceived stress and cortisol awakening response in humans

Considering that the gut microbiota has been implicated in the modulation of host mood and food intake behaviour [10,64], we investigated whether *B. longum* APC1472 could improve levels of the stress hormone cortisol upon waking (i.e. cortisol awakening response), or self-reported measures of satiety, and self-reported measures of mood (i.e. perceived stress, anxiety and depression) (Table 3). *B. longum* APC1472 did not impact cortisol awakening response, or self-reported satiety, perceived stress, anxiety and depression measures.

**Table 3:** Overview of satiety, mood, perceived stress and cortisol awakening response data in human subjects.

	Placebo (*n* = 48)				*B. longum* APC1472 (*n* = 74)				Placebo vs *B. longum* APC1472		
Variable	Week 0	Week 6	Week 12	P-value Week 0–12	Week 0	Week 6	Week 12	P-value Week 0–12	Difference (95% CI)	P-value	η2
**Questionnaire data**											
**Perceived stress** **(Cohens PSS)**	11.2 ± 0.8	10.4 ± 0.8	10.6 ± 0.9	0.422	11.8 ± 0.6	10.6 ± 0.7	10.3 ± 0.8	0.013	−0.859 (−2.7 to 1.0)	0.354	0.007
**Depression (HADS)**	2.45 ± 0.34	2.44 ± 0.35	2.13 ± 0.32	0.315	2.66 ± 0.32	2.57 ± 0.36	2.20 ± 0.32	0.073	−0.089 (−0.82 to 0.64)	0.809	<0.001
**Anxiety (HADS)**	4.54 ± 0.44	4.40 ± 0.47	4.31 ± 0.54	0.572	4.76 ± 0.41	4.65 ± 0.46	4.35 ± 0.49	0.237	−0.154 (−1.21 to 0.90)	0.772	0.001
**Hunger/Satiety**	5.00 ± 0.41	4.96 ± 0.41	6.23 ± 0.44	0.026	5.09 ± 0.30	5.86 ± 0.32	5.53 ± 0.30	0.195	−0.649 (−1.60 to 0.30)	0.181	0.015
**Cortisol awakening response**											
**AUCi (nmol/L)**	3.22 ± 7.20	/	9.41 ± 4.37	0.967	1.29 ± 3.26	/	3.55 ± 4.39	0.725	−6.514 (−17.29 to 4.26)	0.233	0.016
**AUC (nmol/L)**	33.3 ± 2.8	/	37.4 ± 3.8	0.155	45.79 ± 3.92	/	43.5 ± 3.3	0.961	1.12 (−9.5 to 11.8)	0.835	0.001
**Average (nmol/L)**	8.6 ± 4.5	/	11.6 ± 2.0	0.271	11.6 ± 1.0	/	10.5 ± 0.8	0.401	0.09 (−2.4 to 2.6)	0.947	<0.001

### 5.10 *B. longum* APC1472 improves fasting glucose levels, active ghrelin and cortisol awakening response in obese individuals

**Fig. 6:**
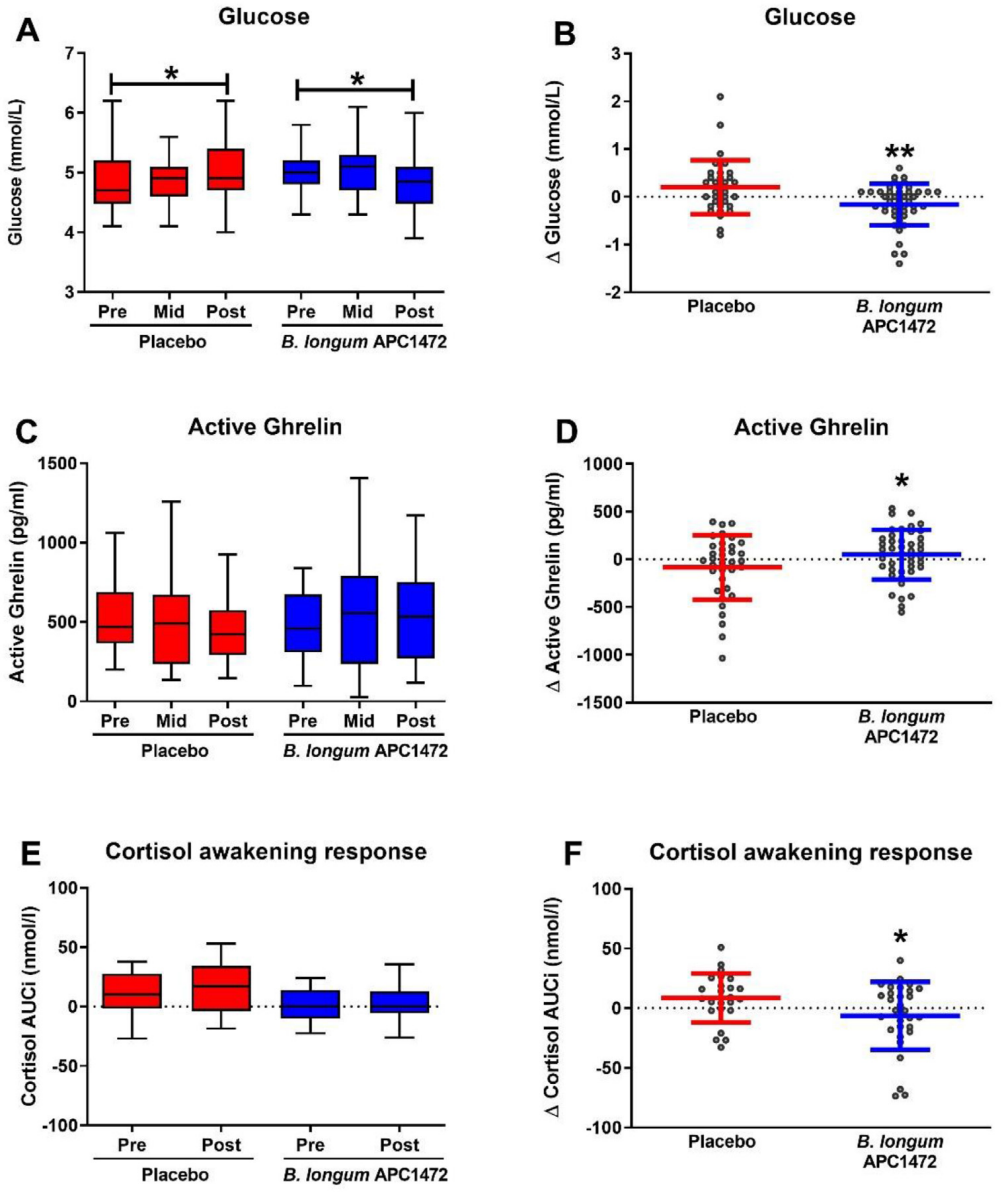
*B. longum* APC1472 supplementation reduces fasting blood glucose levels and cortisol awakening response and increase active ghrelin in obese individuals. Fasting glucose and active ghrelin levels were measured at the beginning of the study (pre), after 6 weeks (mid) and after 12 weeks (post) of treatment. Cortisol awakening response was only assesed at the beginning of the stduy. All data are depicted of all 3 timepoints (A, C, E), as well as the change after 12 weeks compared to at the beginning of the study (B, D, F). Data are depicted as boxplot or scatter dot plot, where the dots depict individual datapoints, with *n* = 36 for the placebo group and *n* = 46 for the *B. longum* APC1472 treatment group. * indicates a significant effect (*p<0.05, **p<0.01).

Participants in this study were either overweight (*n* = 40; 28 ≥ BMI < 30) or obese (*n* = 82; 30 ≥ BMI < 35). It is possible that *B. longum* APC1472 may evoke a stronger effect in obese individuals as they have a stronger phenotype compared to overweight individuals. As such, we investigated whether any of the anthropomorphic measures, blood biomarkers and measures of mood were affected by *B. longum* APC1472 in the obese subpopulation only, compared to placebo (Fig. 6 and **Table S7–11** for population characteristics and full statistical results). Similar to the analysis on the entire study population, *B. longum* APC1472 and placebo reduced fasting glucose levels over the 12-week treatment period (Fig. 6**A**). However, glucose levels were 0.295 mmol/L (95% CI [−0.5, −0.1]) lower in the *B. longum* APC1472 group compared to the placebo group (*F*(1,75) = 7.566, *p* = 0.007), in obese individuals, with a moderate effect size (η^2^ = 0.092) (Fig. 6**B**). Furthermore, *B. longum* APC1472 increased active ghrelin levels (*F*(1,74) = 4.903, *p* = 0.030), with a moderate effect size (η^2^ = 0.062). Moreover, *B. longum* APC1472 also reduced cortisol awakening response (*F*(1,51) = 4.415, *p* = 0.041), with a moderate effect size (η^2^ = 0.080), in the obese subpopulation analysis.

Overall, these results show beneficial effects of *B. longum* APC1472 on fasting plasma glucose levels, active ghrelin levels and cortisol awakening response in obese individuals. It is also important to note that the effect size in the obese subpopulation (η^2^ = 0.092) was bigger than the effect size in the overall study population (η^2^ = 0.075). This indicates that *B. longum* APC1472 has a more robust beneficial effect on fasting glucose levels in obese, rather than in overweight, individuals.

### 5.11 *B. longum* APC1472 does not induce major rearrangements on the microbiota composition but increases the abundance of *Bifidobacterium*

We subsequently investigated whether the observed changes induced by the *B. longum* APC1472 strain were mediated in part through modulation of the gut microbiota. Investigations into the caecal microbiota in the preclinical experiment revealed that there was a significant dissimilarity in beta diversity between LFD- and HFD-fed mice (*p* < 0.01) (**Figure S5A**), with a decreased relative abundances of *Bacteroidetes* phylum and increased relative abundances of *Firmicutes* class *Clostridia*, respectively (**Figure S5B**), which is in line with previous studies [65,66]. Different phylotypes were responsible for the caecal microbiota differences amongst the treatment groups (**Figure S5C**), showing increments on different *Firmicutes* members in HFD-fed mice treated with *B. longum* APC1472. Moreover, *B. longum* APC1472 partially ameliorated the HFD-induced decrease in *Bifidobacteriaceae* relative abundance (*p* = 0.054, adjusted *p* = 0.170) (**Figure S5D**).

**Fig. 7:**
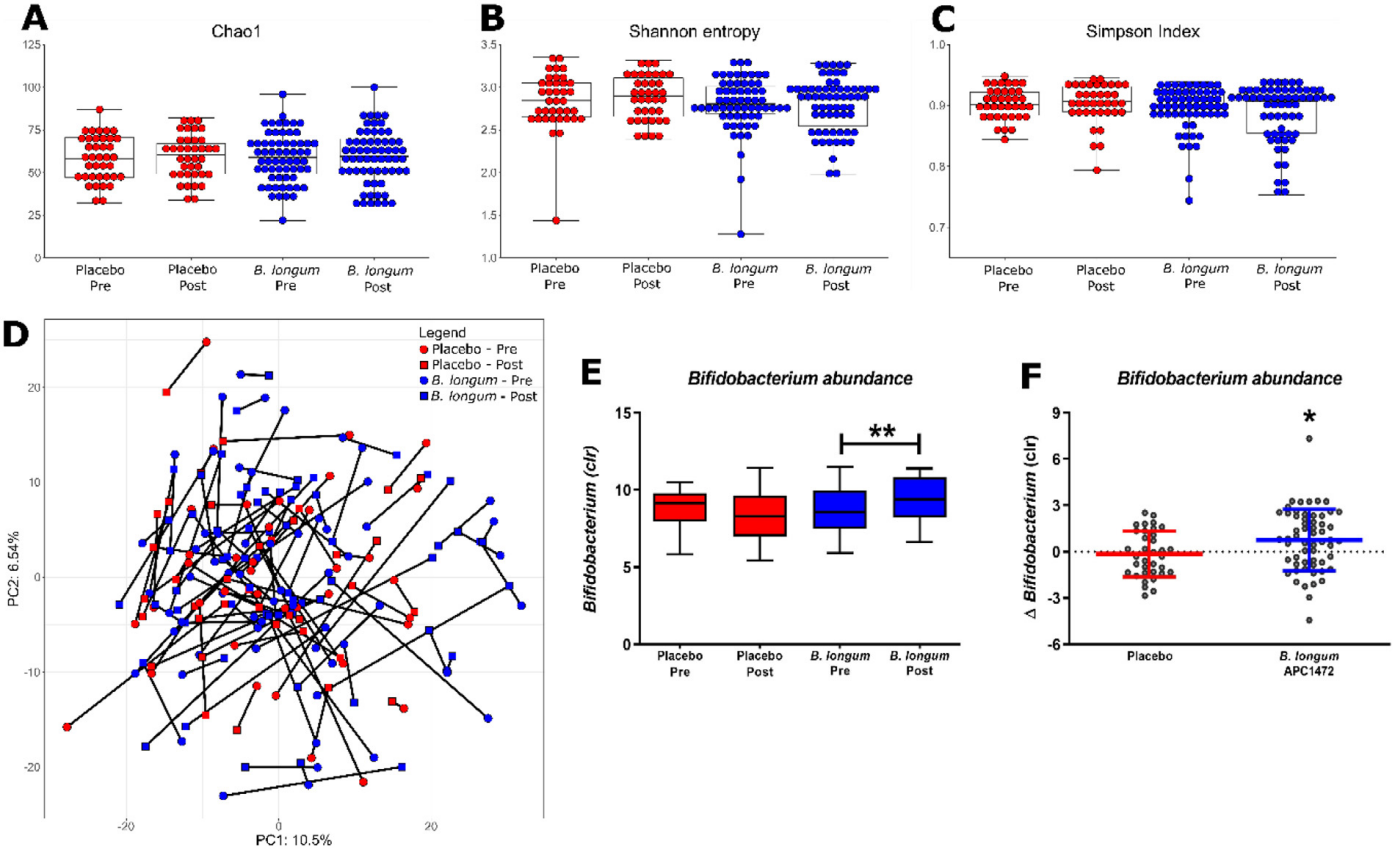
*B. longum* APC1472 increases *Bifidobacterium* abundance without impacting the overall composition of the gut microbiota in humans. The gut microbiota was assesed at the beginning (pre) and end of the study (12 weeks, past). Alpha (A-C) and beta diversity (D) were investigated, as wel as the bacterial genera present (E-F). Microbial taxa were centre-log-transformed (CLR). Significant differences between pre and post were anlysed using the Mann-Whitney U test, whereas treatment differences were analysed using an ANCOVA controlling for sex and pre-intervention *Bifidobacterium* abundance. Data are depicted as boxplot or scatter dot plot, where the dots depict individual datapoints, with *n*= 48 for the placebo group and *n* = 74 for the *B. longum* APC1472 treatment group. * indicates a significant effect (*p<0.05, **p<0.01).

Analysis of the faecal microbiota in the human intervention study revealed that *B. longum* APC1472 did not impact the alpha diversity indices (Shannon, Simpson and Chao1**,** Fig. 7**A-C**). Furthermore, the overall composition of the microbiota remained unaffected as determined by the PCA analysis of the beta diversity (Fig. 7**D**). *B. longum* APC1472 did increase *Bifidobacterium* relative abundance over the 12-week intervention period (t(57) = −2.891, *p* = 0.005), which was not observed in the placebo group (Fig. 7**E**). This resulted in a higher *Bifidobacterium* abundance in the treatment group compared to the placebo group post-intervention (F(3, 89) = 5.922, *p* = 0.017) (Fig. 7**F**). Similar results were observed in the obese subpopulation (**Figure S6**).

Short-chain fatty acids (SCFAs) are potentially one of the most investigated gut microbiota-derived metabolites implicated in host energy metabolism and obesity symptomatology [10,67]. Analysis of faecal SCFA levels in human samples revealed no differences in levels of acetate, propionate, butyrate and valerate (**Table S6**). Furthermore, isobutyrate and isovalerate levels remained unaffected (**Table S6**).

## 6 Discussion

There has been an increased emphasis on gut microbiota-targeted therapeutics for the amelioration of obesity [4,11,68,[69], [100]]. For example, recent studies have identified several probiotic strains with different anti-obesity effects, including members of the genus *Bifidobacterium* [4,[30], [31], [32], [33], [34], [35], [36]], but the exact mechanisms of action are still lacking. In the present study, we demonstrate that a novel isolated *B. longum* APC1472 strain, which was previously shown to attenuate ghrelinergic signalling [37], reduces body weight gain, fat depot size, glucose tolerance and leptin levels in a preclinical mouse model of HFD-induced obesity. Furthermore, when the *B. longum* APC1472 strain was investigated in a human cohort of healthy overweight and obese individuals, a reduced fasting blood glucose level was observed. Noteworthy, stratification and analysis of the obese human subpopulation revealed that *B. longum* APC1472 was able to normalize active ghrelin levels and the cortisol awakening response, which are both dysregulated in obesity [44,[70], [71], [72], [73], [74]]. This highlights the translational value of this novel *Bifidobacterium longum* species, *B. longum* APC1472, from a preclinical mouse model to a human intervention study where this probiotic positively impacts markers of obesity, which may be linked to the ghrelinergic effects previously demonstrated [37]. Specifically, we found that in the preclinical mouse model of obesity, the supplementation with *B. longum* APC1472 significantly reduced fat depots and body weight gain in HFD-fed mice independent of energy intake. Furthermore, *B. longum* APC1472 significantly reduced circulating leptin levels in HFD-fed mice, which is in line with the reduction in fat depot size as leptin is released into the bloodstream in proportion to body fat mass [75]. Notably, circulating levels of leptin were increased in HFD-fed mice compared to LFD-fed mice with no alterations in leptin receptor hypothalamic expression, suggesting no alterations in leptin sensitivity, as has been previously reported in obesity [76]. No changes were observed in the hypothalamic expression of the orexigenic peptides *NPY* and *AgRP* following *B. longum* APC1472 supplementation in mice. Both *NPY* and *AgRP* are orexigenic peptides that increase food intake when overexpressed or when administered centrally [77,78] and HFD-fed mice demonstrate, as expected, a decrease in both of these orexigenic peptides. In contrast, increased hypothalamic expression of anorexigenic peptides such as *POMC* and *CART* in response to a high-fat diet has been suggested as a natural feedback mechanism in order to maintain energy balance and body weight homeostasis [79,80]. The *B. longum* APC1472 was able to normalize the increased hypothalamic expression of the anorexigenic peptide *CART* in HFD-fed mice, suggesting a lower degree of energy imbalance and, therefore, a potential reduced metabolic dysfunction compared to HFD-fed mice. Moreover, *CART* is regulated by leptin and its expression is positively correlated with leptin levels [81]. Therefore, the decreased leptin levels observed in the *B. longum* APC1472-HFD group also support the observed decreased *CART* expression. This highlights the potential of *B. longum* APC1472 to modulate hypothalamic gene expression involved in energy homeostasis and appetite regulation, which warrants further investigation.

In the human intervention study, no difference was observed in the primary outcome of BMI, even though the *B. longum* APC1472 supplementation was able to reduce body weight gain in HFD-induced obese mice. Similarly, no difference was observed in the supportive secondary outcome W/H ratio. This discrepancy might be explained by the fact that the majority of the human intervention cohort was non-diabetic, whereas the HFD-induced obese mice had a decreased glucose tolerance, implying that host glucose metabolism may have been the main factor driving the reduction in body weight gain in the obese mice. It must also be noted that the treatment duration of the preclinical study was longer and, therefore, a longer treatment period in the human intervention study, or a higher treatment dosage, could have resulted in more significant differences and bigger effect-sizes. The 12-week duration of the human study may have been too short of a time to see significant changes in BMI and W/H ration. In addition, using a mixture of bacterial strains, including *B. longum* APC1472, might result in a higher treatment efficacy, as some evidence suggests that multi strain probiotics may be more effective [85]. Age has also been shown to affect body fat distribution and metabolism increasing both the risk and the severity of obesity development [82]. Therefore, some of the discrepancies and lack of translation between the mice study and humans could be explained by the relatively low age of the mice (adolescence to adulthood) versus the human cohort with an average age at midlife. A low age may facilitate a better response to changes in metabolic and physiologic responses and therefore a higher capacity to positively respond to therapeutic interventions. Moreover, the administration strategies were differences between both studies. The mouse study followed a prevention strategy as *B. longum* APC1472 was administered before obesity was established, while in the human study the participants were already obese at the time of administration and, therefore, presented a more severe condition to ameliorate.

Most notably, the *B. longum* APC1472 supplementation significantly improved glucose tolerance in HFD-induced obese mice. Similarly, *B. longum* APC1472 decreased fasting blood glucose levels in overweight/obese individuals (−0.266 mmol/L compared to placebo). It is important to note that the participants in this study had average fasting blood glucose levels of 5.0 mmol/L, which is considered healthy and non-diabetic (*n* = 11 were prediabetic). Above 5.6 mmol/L is considered prediabetic, whereas above 6.9 is considered diabetic [83,84]. These data indicate that *B. longum* APC1472 may have a bigger effect-size on fasting blood glucose levels in a prediabetic or diabetic population, which warrants further investigations. This is further reinforced by the obese subpopulation analysis of the obese individuals, rather than overweight and obese combined, which revealed a fasting blood glucose level (−0.295 mmol/L compared to placebo), which constitutes a bigger effect-size in fasting blood glucose levels (η^2^ = 0.092 vs 0.075), indicating a more potent treatment efficacy in obese individuals. This warrants further investigation into the effect of *B. longum* APC1472 in a cohort of prediabetic or diabetic individuals.

The underlying mechanisms for the decreased fasting blood glucose levels may be associated with the changes in ghrelinergic signalling, as *B. longum* APC1472 was found to attenuate ghrelinergic signalling *in vitro* [37] and ghrelin has been shown to be involved in glucose homeostasis via inhibition of insulin secretion [85]. Moreover, insulin receptor substrate 1 (IRS-1) has been reported to play a key role in glucose homeostasis being involved in glucose transporter 4 (GLUT-4) mobilization [86,87]. Low IRS-1 expression levels have been associated with glucose and insulin sensitivity impairments [86,87]. Therefore, increased IRS-1 expression in epididymal fat tissue of *B. longum* APC1472 treated mice may have also influenced glucose homeostasis. Nevertheless, glucose metabolism is multifactorial and other mechanisms are likely also affected following the supplementation of the *B. longum* APC1472. However, while the biggest effect-size was observed on plasma glucose levels in both the preclinical and human intervention studies, it is also possible that the other observed effects are secondary to the decrease in plasma glucose levels.

Notably, obesity is associated with decreased circulating levels of ghrelin [44,72], which we also observed in the HFD-fed mice and the reason why the ghrelinergic system has been implicated as a promising therapeutic target to combat obesity [45,88]. Indeed, the “hunger hormone” ghrelin was first described as a growth hormone secretagogue, but its key role in the regulation of appetite, food intake, adiposity and metabolism have directed the main therapeutic focus of ghrelin and its receptor towards obesity research with promising anti-obesity potential [41,45,76,[89], [90], [91], [92]]. Interestingly, *B. longum* APC1472 supplementation increased levels of active ghrelin, but not total ghrelin levels, in healthy obese individuals. The increase in active ghrelin may indicate an amelioration of the deficiencies in ghrelinergic signalling associated with obesity. It is also interesting to note that *B. longum* APC1472 was selected on its ability to modulate the ghrelinergic system *in vitro* [37]. Future studies are warranted to investigate if administration of other bacterial strains and their metabolites, including SCFAs, which equally showed the ability to modulate ghrelin signalling *in vitro* [37], have similar effects in obese individuals.

Furthermore, our data reveal that *B. longum* APC1472 decreased fasting corticosterone levels in HFD-induced obese mice, indicating the downregulation of the hypothalamic-pituitary-adrenal (HPA) axis. In line with these results, *B. longum* APC1472 reduced cortisol awakening responses in obese individuals. Dysregulation of the HPA axis, which is colloquially seen as the “body's stress system”, is a risk factor for obesity-related conditions such as cardiovascular disease, insulin resistance and type 2 diabetes [93]. Hence, the stress hormone cortisol (corticosterone in rodents), which is central in the HPA axis, has been shown to promote the accumulation of fat cells and weight gain [93] and to regulate the function of pancreatic α and β cells affecting glucagon and insulin secretion [94]. As such, even though no changes were observed in insulin, the changes in cortisol awakening responses could indicate that the HPA axis has contributed to the *B. longum* APC1472-induced decrease in fasting blood glucose. Furthermore, the HPA axis is also affected by ghrelin, indicating that the observed changes in ghrelin could have also contributed to the changes in cortisol [95], [99].

Finally, we investigated the effects of *B. longum* APC1472 treatment on gut microbiota composition. Overall, *B. longum* APC1472 treatment did not have a major impact on microbiota composition other than the partial restoration of *Bifidobacterium* levels in HFD-fed mice. These findings are in line with the effects of *B. longum* APC1472 on healthy human overweight and obese individuals and with other investigations on obesity using different probiotics strains, where major rearrangements on microbiota composition were also not observed [96,97].

Of note, while the modulation of ghrelin receptor signalling by *B. longum* APC1472 strain may have contributed to an improved metabolic profile, we cannot rule out other beneficial anti-obesity effects. As such, future studies are warranted further investigating the mechanisms and metabolites through which *B. longum* APC1472 modulates host glucose homeostasis, with a focus on the ghrelinergic system.

In conclusion, we have demonstrated positive anti-obesity effects of the novel *B. longum* APC1472 strain in HFD-induced obese mice and a partial translation of these positive effects of *B. longum* APC1472 supplementation in otherwise healthy overweight and obese individuals. In particular, we show the promising potential of *B. longum* APC1472 to be developed as a valuable supplement in reducing specific markers of obesity, possibly via the ghrelinergic system. Most notably, the decrease in fasting plasma glucose induced by *B. longum* APC1472 may have clinically significant health implications for prediabetic and type 2 diabetes mellitus populations in particular.

## Data sharing

Deidentified data and related documents will be made available upon request.

## Disclosure

This research was funded in part by Science Foundation Ireland in the form of a Research Centre grant (SFI/12/RC/2273) to APC Microbiome Ireland and by a research grant from Cremo S.A. J.F.C and T.G.D have research support from Mead Johnson, Cremo, 4D Pharma, Dupont, and Nutricia. J.F.C, T.G.D and P.D.C. have spoken at meetings sponsored by food and pharmaceutical companies. All other authors report no conflicts of interest.

## Contributions

H.S. contributed to the design of the study, interpreted the data and ledthe writing of the manuscript. C.T.F. contributed to the design of the preclinical study, performed the preclinical study and contributed to the writing of the manuscript of the preclinical study. M.vdW. performed ELISAs, MSD assays, interpreted the data and contributed to the writing of the manuscript of the human intervention study. C.M.L.S. designed the human intervention study and interpreted the data and contributed to the writing of the manuscript. Avery M. performed DNA isolations, sequenced the DNA and assisted with the SCFA quantifications of the human intervention study. Amy M. assisted with the DNA isolations of the human intervention study. C.S. performed the SCFA quantifications of the human intervention study. K.B. interpreted the nutritional data. T.F.S.B. and F.F. performed the bioinformatics for the microbiota for the human intervention study. K.R. interpreted the data and contributed to writing the manuscript. A.G. assisted with running the preclinical study. S.A. performed library preparation and SCFA quantifications of the preclinical study. K.M. performed the bioinformatics of the preclinical study. M.V.P assisted with running the preclinical study. M.M.P. performed the corticosterone quantifications. P.R., B.L.R., C.S., T.G.D. and J.F.C. contributed to the design of the study, interpreted the data and contributed to the manuscript. Finally, T. G. D was Chief Investigator on the clinical part of the study.

## Appendix Supplementary materials

Image, application 1
