# Supplementary material for: Bifidobacterium longum counters the effects of obesity: Partial successful translation from rodent to human
Source: eBioMedicine. 2020 Dec 18;63:103176. doi: 10.1016/j.ebiom.2020.103176 (PMC7838052; doi:10.1016/j.ebiom.2020.103176)
Supplement: Supplementary file 1 [file mmc1.docx]

Dr. Harriet Schellekens, Department of Anatomy and Neuroscience / Alimentary Pharmabiotic Centre (APC) Microbiome Ireland, University College Cork, Cork, Rep. of Ireland. Email: H.Schellekens@ucc.ie Tel.: +353 21490 5429

**Supplementary information**

***Bifidobacterium longum* Counters the Effects of Obesity: Partial Successful Translation from Rodent to Human**

Harriët Schellekens***^£^**^1,2^, Cristina Torres-Fuentes*^1^, Marcel van de Wouw*^1^, Caitriona M Long-Smith^1^, Avery Mitchell^3^, Conall Strain^3^, Kirsten Berding^1^, Thomaz F. S. Bastiaanssen^1,2^, Kieran Rea^1^, Anna V. Golubeva^1,2^, Silvia Arboleya^1,3^, Mathieu Verpaalen^1,2^, Matteo M. Pusceddu^1^, Amy Murphy^1,3^, Fiona Fouhy^1,3^, Kiera Murphy^3^, Paul Ross^1,4^, Bernard L. Roy^5^, Catherine Stanton^1,3^, Timothy G. Dinan^1,6^ and John F.Cryan**^£^**^1,2^

^1^APC Microbiome Ireland, University College Cork, Cork, Ireland

^2^Department of Anatomy and Neuroscience, University College Cork, Cork, Ireland

^3^Teagasc Food Research Centre, Moorepark, Fermoy, Cork

^4^College of Science Engineering & Food Science, University College Cork, Cork, Ireland

^5^Cremo SA, Villars-sur-Glâne, Fribourg, Switzerland

^6^Dept of Psychiatry, University College Cork, Cork, Ireland

* Equal contributing authors; **£** joint senior authors

Corresponding author: H.Schellekens@ucc.ie

***B. longum APC1472* survival in drinking water**

*Bifidobacterium longum* APC1472 survival analysis in drinking water was carried out by examination of viable counts using *Bifidobacterium* selective agar medium. Briefly, *Bifidobacterium longum* APC1472 was grown anaerobically in MRS medium and bacterial pellet was then dissolved in drinking water as described in the material and methods section for mouse study administration. Aliquots were then collected at different time points, diluted and plated on MRS agar supplemented with cysteine hydrochloride (0.05% w/v) and mupirocin (100 µg/ml) (Oxoid, UK). Samples were incubated under anaerobic conditions at 37°C for 48 h. Colonies were counted and results were expressed as CFU per mL accordingly to the corresponding dilution factor.

**Fecal Bifidobacterium quantification**

Examination of viable counts of Bifidobacterium in stools was performed by using *Bifidobacterium* selective agar medium. Briefly, fresh fecal pellet were weighed, placed in an sterile Eppendorf tube and homogenized in sterile PBS (10% w/v). MRS agar supplemented with cysteine hydrochloride (0.05% w/v) and mupirocin (100 µg/ml) (Oxoid, UK) was used for Bifidobacterium CFU quantification. Samples were incubated under anaerobic conditions at 37°C for 48 h. Colonies were counted and results were expressed as CFU per gram accordingly to the dilution and fecal pellet weight.

### Human faecal sample collection

The majority of the faecal samples were egested in the morning of the visit (73%), whereas the rest was egested the day before (26%), but within 12 hours of the study visit. An AneroGen sachet (Oxoid, Thermo Scientific) was included in the sampling kit. Participants were instructed to keep faecal samples in the fridge until delivery at the visit time. Samples were immediately frozen at -80 °C at the visit. Of each sample, 200 mg was taken from the core after thawing and subsequently used for DNA extractions and 16s sequencing, and another 200 mg aliquot was used for SCFA quantifications.

### Human faecal DNA extraction

Samples were homogenised with lysis buffer (NaCl, Tris-HCl and SDS) in a clean microtube with Zirconia/silica beads (0.4 g of 0.1 mm beads, 0.4 g of 1.0 mm beads and 0.2 g of 2.3 mm beads, Biospec) for 3 minutes using a bead beater (MiniBeadBeater, Biospec). Samples were subsequently incubated at 70 °C for 15 minutes, which was followed by centrifugation. The remaining pellet underwent a second lysis buffer homogenisation/ bead beating step followed by heat treatment and centrifugation. Both supernatants were pooled, mixed with 10M ammonium acetate and incubated for at 5 °C for 5 minutes, which was followed by centrifugation. The remaining supernatant was mixed with an equal part of 2-propanol and incubated at -20 °C for 30 minutes. Samples were subsequently centrifuged, and supernatants were discarded. The remaining pellet was washed in 70% ethanol, allowed to dry, and dissolved in Tris-EDTA buffer (ATE). The dissolved pellets were incubated with RNAse at 37 °C for 15 minutes and were then incubated with proteinase K at 70 °C for 10 minutes, after which 200 µL of ethanol was added. Samples were loaded onto spin columns and washed with wash buffers AW1 followed by AW2, as performed according to the manufacturer’s instructions. The columns were spun dried before eluting in ATE buffer and DNA samples were stored at -30 °C.

**Faecal SCFA quantification**

Homogenised samples were subsequently centrifuged and filtered using a 0.2 μm syringe filter (Corning). A 30 µL aliquot of the internal standard (10 mM) was added to 270 µL of each supernatant and vortexed for 10 seconds. Samples were centrifuged (Mikro 200R, Hettich) at 26,000 g for 2 minutes at 4 °C. Supernatants were transferred into a 250 μl glass insert and placed in a 2 mL amber GC vial and sealed with a PTFE cap (Agilent).

A 7-point standard curve was generated with: Acetate (0.1 mM – 10 mM), propionate (0.1 mM – 10 mM), IsoButyrate (0.01 mM – 1 mM), Butyrate (0.1 mM – 10 mM), IsoValerate (0.01 mM – 1 mM), and valerate (0.1 mM – 10 mM) in acidified water (pH 3; HCl). 2-Ethylbutyric acid (1 mM) was used as an internal standard. Standards and samples were analysed.

### Figure S1. *B. longum* APC1472 concentrations and impact on water and caloric intake.


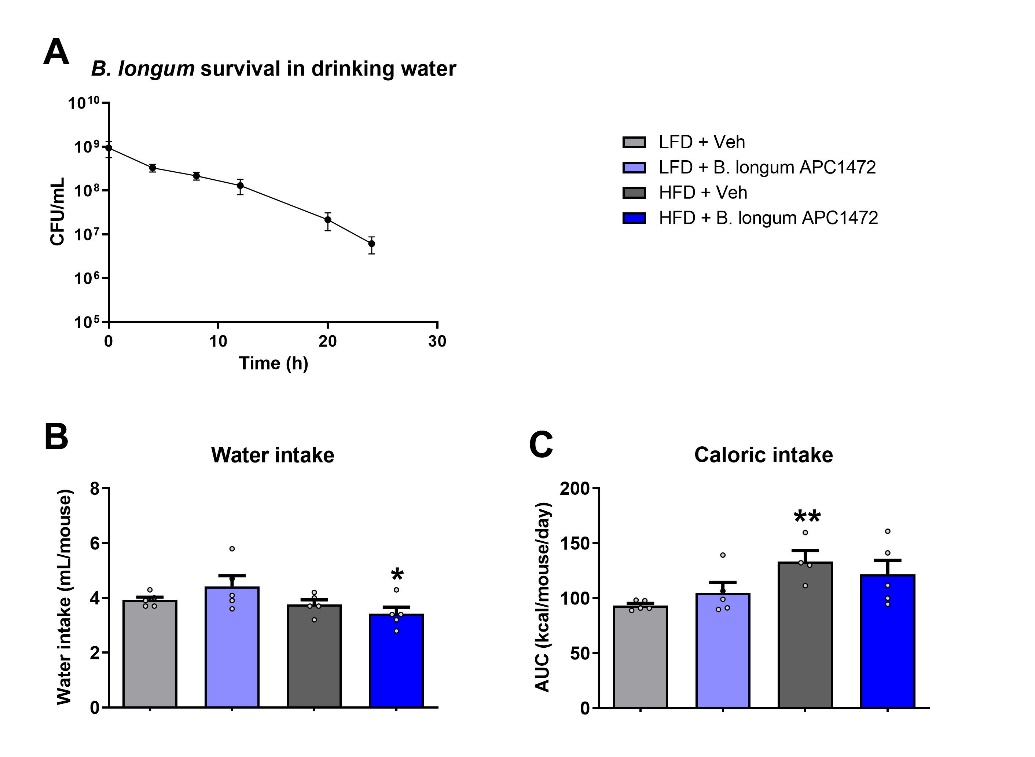


**Figure S1.** ***B. longum* APC1472 concentrations and impact on water and caloric intake.** (A) The concentration of *B. longum* APC1472 was measured over 24 hours in the drinking water of mice. (B, C) Water and caloric intake were measured in all experimental groups. Data are shown as means ± SEM. Data are significant different (p<0.05) accordingly to two-way ANOVA followed by LSD post-hoc. * indicates significant difference in HFD compared to LFD (*p<0.05, **p<0.01).

### Figure S2. Human intervention study outline.


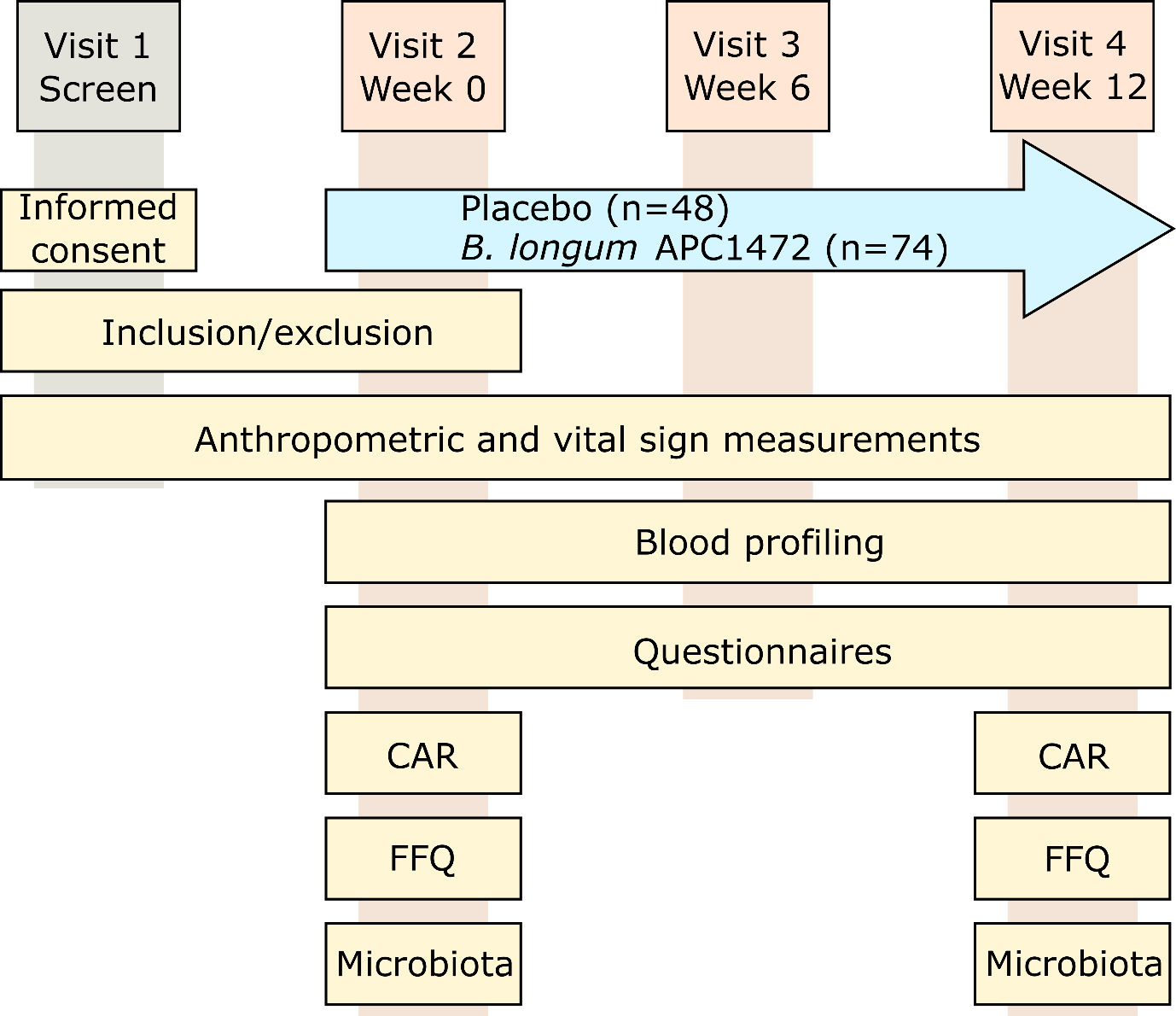


Figure S2. Human intervention study outline. On visit 1, participants were assesed for their eligibility to participate in the study and explain which procedures would be undertaken. On visit 2, baseline measurements were taken and participants were asked to start taking the study product (i.e. placebo or Bifidobacterium longum APC 1472). These measurements were re-assesed after 6- and 12-weeks. Abbreviations: CAR = cortisol awakening response, FFQ = Food frequency questionaire.

### Figure S3. *B. longum* APC1472 treatment significantly decreased orexigenic neuropeptides expression in the hypothalamus of mice.

**
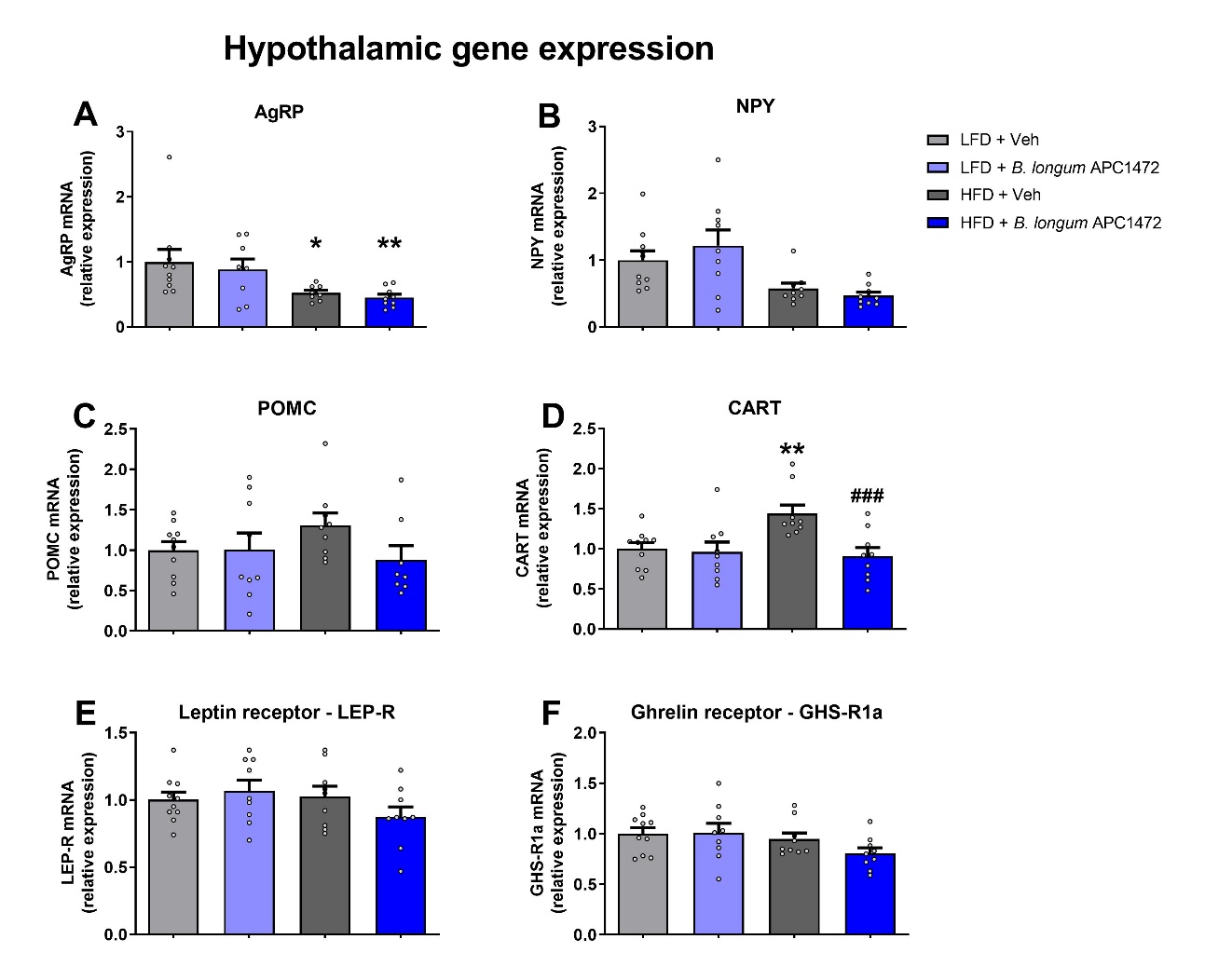
**

**Figure S3. *B. longum* APC1472 treatment significantly decreased orexigenic neuropeptides gene expression in the hypothalamus of mice.** (A) mRNA expression of orexigenic markers agouti-related protein (*AgRP*) and (B) neuropeptide Y (*NPY*), (C) pro-opiomelanocortin (*POMC*), (D) cocaine- and amphetamine-regulated transcript (*CART*), (E) leptin receptor (*LEP-R*) and (F) ghrelin receptor (*GHS-R1a*) measured in the hypothalamus of mice treated with drinking water containing sterile PBS (2% vol/vol) and glycerol (0.5% vol/vol) and fed a control low-fat diet (LFD) (n=9 in A; n=10 in B, C and D) or a high-fat diet (HFD) (n=9) and in mice treated with *B. longum* APC1472 in drinking water (2x10^8^ CFU/mL) and fed a LFD (n=9) or a HFD (n=9) for 16 weeks. Data are shown as means ± SEM. Data are significantly different (p<0.05) according to two-way ANOVA followed by LSD post-hoc test (A, B, C, D, E, F). * indicates a significant difference in HFD compared to LFD (*p<0.05, **p<0.01), ^#^ indicates a significant difference in *B. longum* APC1472 supplemented mice and controls (^#^p<0.05, ^##^p<0.01, ^###^p<0.001)

### Figure S4. HFD decreases total ghrelin but does not impact on triglycerides in mice.


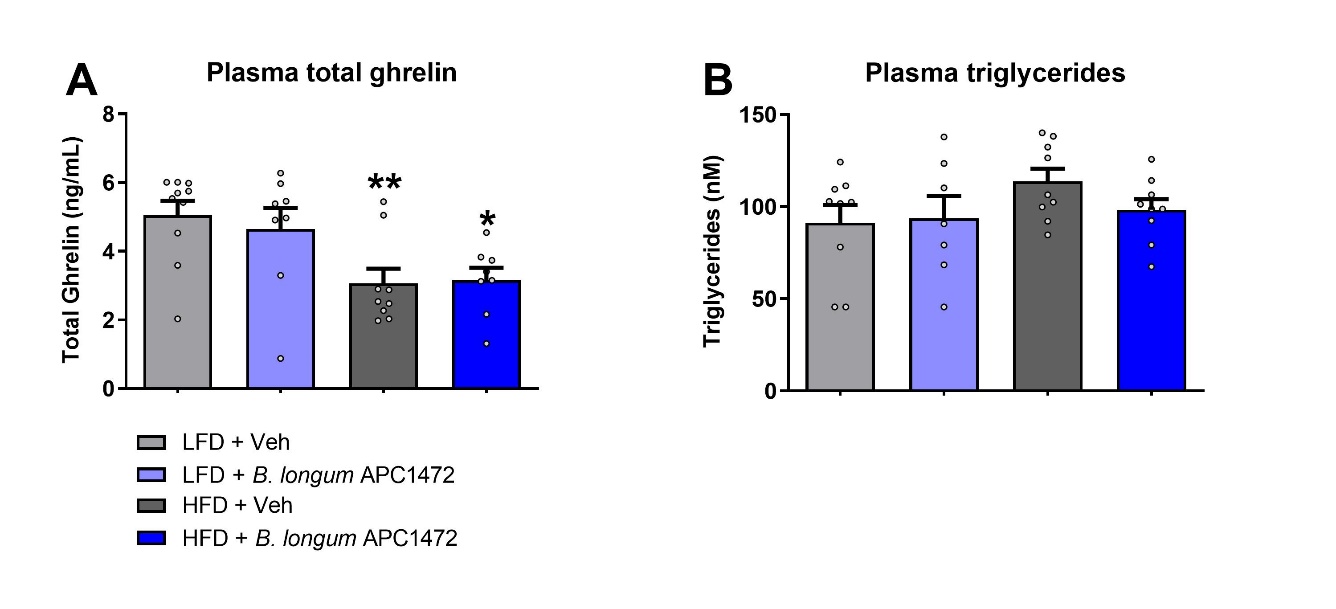


**Figure S4. HFD decreases total ghrelin but does not impact on triglycerides in mice.** (A) plasma total ghrelin and (B) plasma triglycerides were measured in mice treated with drinking water containing sterile PBS (2% vol/vol) and glycerol (0.5% vol/vol) and fed a control low-fat diet (LFD) (n=9 in A; n=10 in B, C and D) or a high-fat diet (HFD) (n=9) and in mice treated with *B. Longum* APC1472 in drinking water (2x10^8^ CFU/mL) and fed a LFD (n=9) or a HFD (n=9) for 16 weeks. Data are shown as means ± SEM. Data are significant different (p<0.05) accordingly to two-way ANOVA followed by LSD post-hoc test (A and B). * indicates significant difference of HFD compared to LFD (*p<0.05, **p<0.01).

### Figure S5. *B. longum* APC1472 effects on caecal microbiota composition in mice.


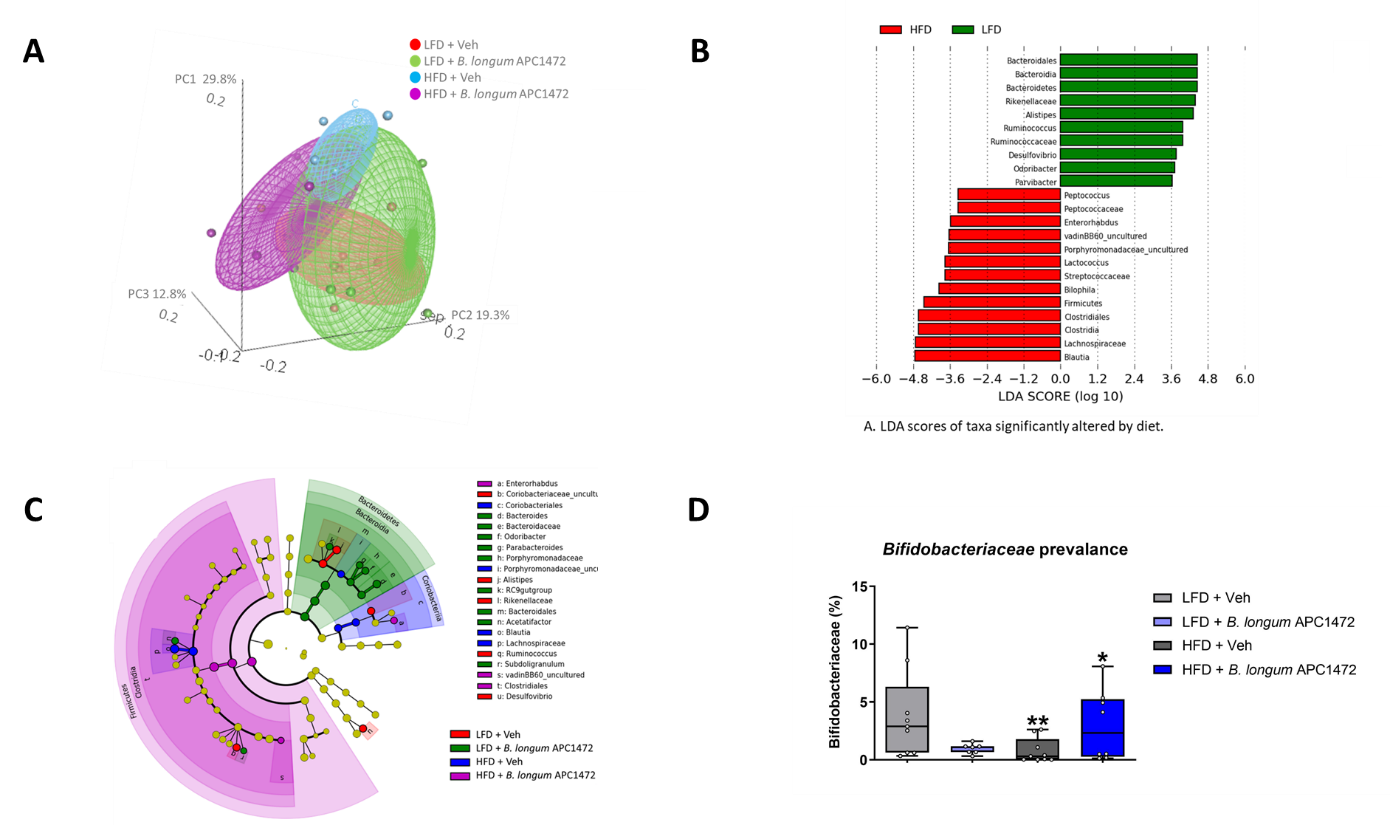


Figure S5. *B. longum* APC1472 effects on caecal microbiota composition in mice. (A) Principal Coordinates Analysis (PCoA) of caecal microbiota composition based on Bray-Curtis beta diversity distances (each point represent the caecal microbiota of a mouse) performed using the Adonis function in the ‘vegan’ (2.4-3) package for R (version 3.3.1), (B) diet-mediated differences in abundance of bacterial caecal communities at family level as assessed by Lda Effective Size (LEfSe) algorithm, (LDA scores > 2 and significance of p<0.05 as determined by Wilcoxon’s signed-rank test) (C) taxonomic cladogram obtained from LEfSe analysis (LDA scores > 2 and significance of p<0.05 as determined by Wilcoxon’s signed-rank test) showing the most differentially abundance taxa among the different treatments groups and (D) relative abundance of *Bifidobacteriaceae*, from control mice treated with drinking water containing sterile PBS (2% vol/vol) and glycerol (0.5% vol/vol) and fed a control low-fat diet (LFD) (n=9) or a high-fat diet (HFD) (n=9) and in mice treated with B. longum APC1472 in drinking water (2x10^8^ CFU/mL) and fed a LFD (n=8) or a HFD (n=8) for 16 weeks. Data are significant different in (D) (p<0.05) accordingly to Kruskal Wallis test followed by Bonferroni p value correction for multiple comparisons.

### Figure S6. *B. longum* APC1472 does not impact composition of the gut microbiota in the obese subpopulation.


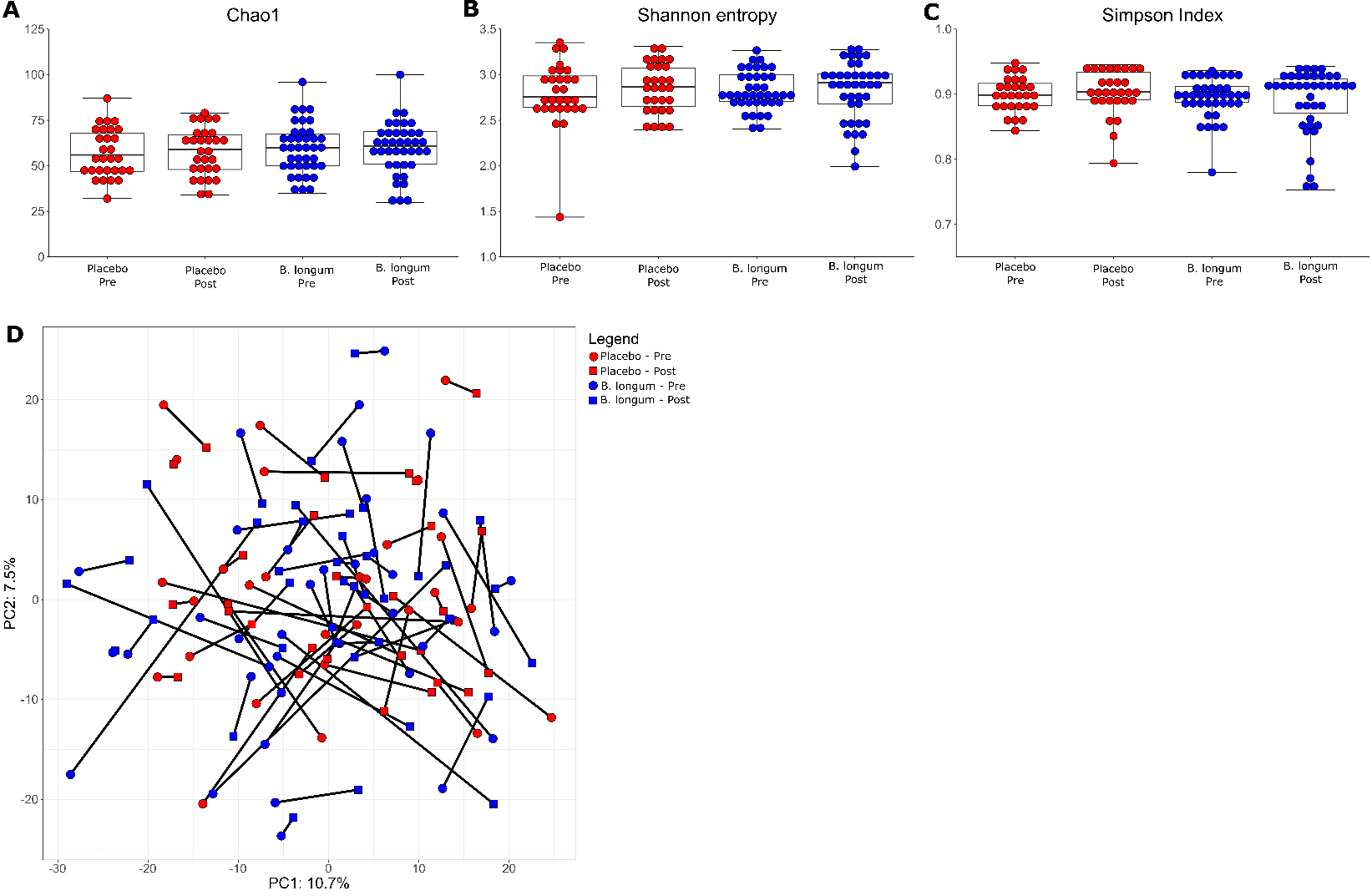


Figure S6. *B. longum* APC1472 does not impact composition of the gut microbiota in the obese subpopulation. The gut microbiota was assesed at the beginning (pre) and end of the study (12 weeks, past). Alpha (A-C) and beta diversity (D) were investigated. Data are depicted as boxplot or scatter dot plot, where the dots depict individual datapoints. N = 48 for the placebo group and n = 74 for the B. longum APC1472 treatment group.

### Figure S7. *B. longum* APC1472 does not impact caecal SCFA levels in HFD mice.


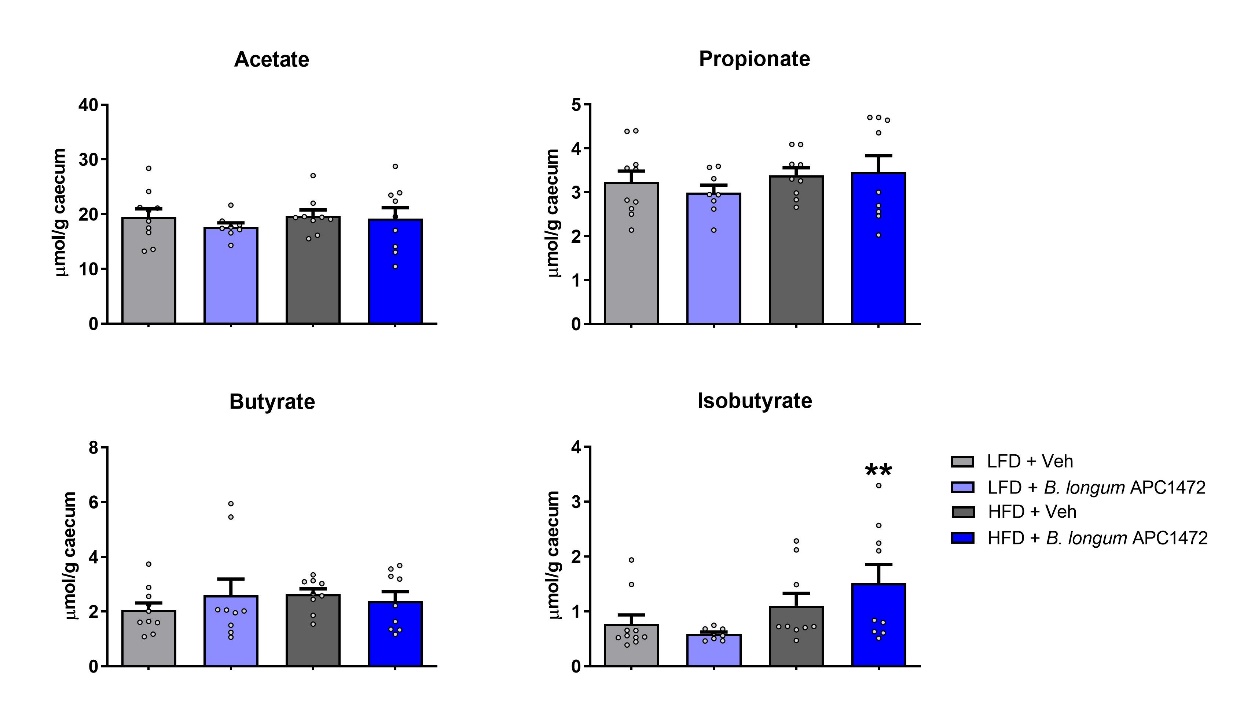


**Figure S7. *B. longum* APC1472 does not impact caecal SCFA levels in HFD mice.** Caecal SCFA levels were quantified from mice treated with drinking water containing sterile PBS (2% vol/vol) and glycerol (0.5% vol/vol) and fed a control low-fat diet (LFD) (n=9 in A; n=10 in B, C and D) or a high-fat diet (HFD) (n=9) and in mice treated with *B. Longum* APC1472 in drinking water (2x10^8^ CFU/mL) and fed a LFD (n=9) or a HFD (n=9) for 16 weeks. Data are significant different (p<0.05) according to a Kruskall-Wallis test followed by Mann-Whitney U test. ^#^ indicates significant difference of LFD-fed mice receiving *B. longum* APC1472 supplementation compared to LFD-fed mice without (p<0.05).

**Table S1 – Primer list**

| Gene symbol | RefSeq ID | IDT Assay ID | Supplier |
| --- | --- | --- | --- |
| *Actb* | NM_007393.1 | Mm00607939_s1 | Applied Biosystems |
| *Npy* | NM_023456(1) | Mm.PT.58.29444574 | Integrated DNA Technologies |
| *Pomc* | NM_008895(1) | Mm.PT.58.5917321 | Integrated DNA Technologies |
| *Cartpt* | NM_001081493(2) | Mm.PT.58.13003479 | Integrated DNA Technologies |
| *Agrp* | NM_001271806(2) | Mm.PT.58.45969609 | Integrated DNA Technologies |
| *Lepr* | NM_001122899(3) | Mm.PT.58.33275723 | Integrated DNA Technologies |
| *Ghsr* | NM_177330.4 | Mm00616415_m1 | Applied Biosystems |

Table S2 Physical activity, caloric and macronutrient intake of participants throughout the study**.**

|  | Placebo (n = 48) | | | *B. longum* APC1472 (n = 74) | | | Placebo vs *B. Longum* APC1472 | |
| --- | --- | --- | --- | --- | --- | --- | --- | --- |
| Variable | Week 0 | Week 12 | P-value  Week 0-12 | Week 0 | Week 12 | P-value  Week 0-12 | Difference  (95% CI) | P-value |
| Physical activity | | | | | | | | |
| Total METS score | 1121 ± 157 | 1211 ± 242 | 0.702 | 2471 ± 977 | 1237 ± 260 | 0.216 | -50.9  (-803 to 701) | 0.893 |
| Food intake profile | | | | | | | | |
| Energy intake (kcal) | 2361 ± 118 | 2248 ± 118 | 0.500 | 2203 ± 89 | 2323 ± 105 | 0.384 | 170.3  (-93 to 434) | 0.203 |
| Carbohydrates (g) | 266 ± 14 | 248 ± 14 | 0.375 | 242 ± 10 | 253 ± 14 | 0.497 | 20.5  (-14 to 55) | 0.244 |
| Of which monosaccharides (g) | 85 ± 5 | 83 ± 6 | 0.807 | 80 ± 4 | 80 ± 5 | 0.904 | 0.1  -14 to 14) | 0.989 |
| Protein (g) | 104 ± 6 | 99.0 ± 4.4 | 0.545 | 95 ± 4 | 99.9 ± 3.6 | 0.415 | 3.8  (-6 to 14) | 0.464 |
| Fat (g) | 94 ± 5 | 91.0 ± 5.6 | 0.708 | 91 ± 4 | 97.0 ± 4.8 | 0.386 | 7.6  (-4 to 19) | 0.198 |
| Of which saturated  fats (g) | 34.8 ± 2.2 | 33.8 ± 2.1 | 0.751 | 34.2 ± 1.8 | 36.4 ± 1.8 | 0.376 | 3.10  (-1.3 to 7.5) | 0.170 |
| Fiber (g) | 27.9 ± 1.4 | 27.0 ± 1.7 | 0.703 | 25.6 ± 1.3 | 26.8 ± 1.5 | 0.538 | 1.15  (-2.6 to 4.9) | 0.545 |

Table S3. Micronutrient intake remains unaltered throughout the study.

|  | Placebo (n = 48) | | | B. longum APC1472 (n = 74) | | | Placebo vs B. Longum APC1472 | |
| --- | --- | --- | --- | --- | --- | --- | --- | --- |
| Variable | Week 0 | Week 12 | P-value  Week 0-12 | Week 0 | Week 12 | P-value  Week 0-12 | Difference  (95% CI) | P-value |
| Monounsaturated fatty acids (g) | 32.9 ± 1.9 | 32.0 ± 2.2 | 0.767 | 32.7 ± 1.6 | 34.2 ± 1.8 | 0.541 | -5.39  (-11.1 to 0.3) | 0.062 |
| Polyunsaturated fatty acids (g) | 14.0 ± 0.7 | 13.6 ± 1.0 | 0.734 | 13.4 ± 0.7 | 14.5 ± 0.9 | 0.297 | -2.76  (-5.5 to -0.1) | 0.045 |
| Saturated fatty acids (g) | 34.8 ± 2.2 | 33.8 ± 2.1 | 0.751 | 34.2 ± 1.8 | 36.4 ± 1.8 | 0.376 | -3.06  (-8.7 to 2.5) | 0.282 |
| Cholesterol (mg) | 370 ± 30 | 338 ± 18 | 0.343 | 328 ± 19 | 331 ± 15 | 0.879 | -5.31  (52.8 to 42.2) | 0.825 |
| Total sugar (g) | 120 ± 7 | 113 ± 7 | 0.494 | 110 ± 5 | 111 ± 6 | 0.928 | -8.93  (-27.7 to 9.9) | 0.348 |
| Fibre (g) | 27.9 ± 1.4 | 27.0 ± 1.7 | 0.703 | 25.6 ± 1.3 | 26.8 ± 1.5 | 0.538 | -1.53  (-6.0 to 3.0) | 0.503 |
| Vitamin A (µg) | 1397 ± 93 | 1475 ± 104 | 0.580 | 1219 ± 85 | 1642 ± 145 | 0.013 | -37.3  (-439 to 364) | 0.854 |
| Thiamine (mg) | 2.0 ± 0.1 | 2.0 ± 1.0 | 0.666 | 1.9 ± 0.1 | 2.1 ± 0.1 | 0.251 | -0.20  (-0.5 to 0.1) | 0.225 |
| Riboflavin (mg) | 2.3 ± 0.1 | 2.2 ± 0.1 | 0.609 | 2.2 ± 0.1 | 2.3 ± 0.1 | 0.273 | -0.01  (-0.3 to 0.3) | 0.965 |
| Niacin (mg) | 46.1 ± 2.9 | 43.8 ± 2.1 | 0.521 | 42.3 ± 1.9 | 44.4 ± 1.6 | 0.402 | -3.12  (-8.3 to 2.1) | 0.235 |
| Pantothenate (mg) | 7.9 ± 0.4 | 7.5 ± 0.3 | 0.452 | 7.3 ± 0.3 | 7.7 ± 0.3 | 0.309 | -0.25  (-1.1 to 0.6) | 0.560 |
| Vitamin B6 (mg) | 2.3 ± 0.1 | 2.3 ± 0.1 | 0.669 | 2.2 ± 0.1 | 2.3 ± 0.1 | 0.416 | -0.14  (-0.4 to 0.2) | 0.352 |
| Vitamin B7 (mg) | 52.6 ± 2.8 | 50 ± 2.5 | 0.566 | 45.9 ± 2.0 | 50.2 ± 2.6 | 0.195 | -2.74  -10.5 to 5.0) | 0.484 |
| Vitamin B9 (µg) | 115 ± 13 | 113 ± 10 | 0.898 | 110 ± 8 | 116 ± 9 | 0.560 | 6.3  (-21 to 33) | 0.646 |
| Vitamin B12 (µg) | 7.9 ± 0.6 | 7.5 ± 0.4 | 0.576 | 7.5 ± 0.4 | 8.4 ± 0.5 | 0.136 | -0.40  (-1.8 to 1.0) | 0.567 |

| Vitamin C (mg) | 144 ± 9 | 140 ± 10 | 0.734 | 132 ± 9 | 132 ± 9 | 0.936 | -16.9  (-44 to 11) | 0.228 |
| --- | --- | --- | --- | --- | --- | --- | --- | --- |
| Vitamin D (µg) | 3.5 ± 0.2 | 3.3 ± 0.2 | 0.637 | 3.1 ± 0.2 | 3.3 ± 0.3 | 0.591 | -0.27  (-1.0 to 0.5) | 0.493 |
| Vitamin E (mg) | 11.1 ± 0.6 | 10.8 ± 0.7 | 0.701 | 10.5 ± 0.5 | 11.1 ± 0.6 | 0.498 | -1.93  (-3.8 to 0.0) | 0.052 |
| Vitamin K1 (µg) | 176 ± 22 | 173 ± 17 | 0.926 | 159 ± 19 | 169 ± 20 | 0.733 | -7.2  (-64 to 50) | 0.804 |
| Sodium (mg) | 2401 ± 151 | 2247 ± 132 | 0.445 | 2242 ± 116 | 2397 ± 130 | 0.376 | -346  (-729 to 37) | 0.076 |
| Potassium (mg) | 4587 ± 183 | 4356 ± 198 | 0.396 | 4217 ± 163 | 4541 ± 211 | 0.227 | -408  (-1007 to 191) | 0.180 |
| Calcium (mg) | 1119 ± 65 | 1090 ± 55 | 0.737 | 1009 ± 45 | 1091 ± 50 | 0.223 | -11.0  (-159 to 137) | 0.883 |
| Magnesium (mg) | 393 ± 21 | 375 ± 19 | 0.516 | 347 ± 13 | 372 ± 15 | 0.219 | -22.1  (-70 to 27) | 0.372 |
| Phosphorous (mg) | 1682 ± 91 | 1610 ± 69 | 0.539 | 1527 ± 60 | 1611 ± 57 | 0.311 | -51.3  (-231 to 129) | 0.574 |
| Iron (mg) | 13.3 ± 0.7 | 12.8 ± 0.7 | 0.644 | 12.3 ± 0.5 | 12.8 ± 0.6 | 0.509 | -0.48  (-2.4 to 1.4) | 0.613 |
| Copper (mg) | 1.46 ± 0.08 | 1.39 ± 0.08 | 0.559 | 1.31 ± 0.06 | 1.48 ± 0.08 | 0.107 | -0.137  (-0.38 to 0.11) | 0.264 |
| Zinc (mg) | 12.5 ± 0.8 | 12.0 ± 0.6 | 0.616 | 11.3 ± 0.5 | 12.0 ± 0.4 | 0.305 | -0.56  (-2.0 to 0.9) | 0.435 |
| Chloride (mg) | 4131 ± 245 | 3757 ± 210 | 0.249 | 3858 ± 198 | 4037 ± 216 | 0.543 | -553  (-1184 to 78) | 0.085 |
| Manganese (mg) | 5.1 ± 0.4 | 5.1 ± 0.4 | 0.905 | 4.3 ± 0.2 | 4.5 ± 0.2 | 0.550 | -0.45  (-1.3 to 0.4) | 0.280 |
| Selenium (µg) | 69 ± 5 | 64 ± 4 | 0.367 | 62 ± 3.5 | 62 ± 2.9 | 0.886 | -3.15  (-12.1 to 5.8) | 0.489 |
| Iodine (µg) | 238 ± 17 | 236 ± 13 | 0.937 | 226 ± 12.5 | 224 ± 11 | 0.917 | -11.5  (-21 to 44) | 0.489 |

## **Table S4. Overview of anthropomorphic data and hormones involved in host metabolism, satiety and appetite**.

|  | **Placebo (n = 48)** | | | | ***B. longum* APC1472 (n = 74)** | | | | **Placebo vs *B. Longum* APC1472** | | |
| --- | --- | --- | --- | --- | --- | --- | --- | --- | --- | --- | --- |
| Variable | Week 0 | Week 6 | Week 12 | P-value  Week 0-12 | Week 0 | Week 6 | Week 12 | P-value  Week 0-12 | Difference  (95% CI) | P-value | η^2^ |
| Anthropomorphic data | | | | | | | | | | | |
| Weight (kg) | 87.9 ± 1.7 | 87.9 ± 1.7 | 88.0 ± 1.7 | 0.730 | 89.0 ± 1.3 | 89.3 ± 1.2 | 89.0 ± 1.3 | 0.894 | -0.087  (-1.1 to 1.0) | 0.868 | <0.001 |
| BMI (kg/m^2^) | 31.3 ± 0.3 | 31.3 ± 0.3 | 31.3 ± 0.3 | 0.681 | 30.8 ± 0.2 | 30.9 ± 0.2 | 30.8 ± 0.2 | 0.974 | 0.03  (-0.4 to 0.3) | 0.887 | <0.001 |
| W-H ratio | 0.95 ± 0.01 | 0.94 ± 0.12 | 0.94 ± 0.01 | 0.489 | 0.96 ± 0.01 | 0.95 ± 0.01 | 0.95 ± 0.01 | 0.039 | 0.003  (-0.01 to 0.01) | 0.506 | 0.004 |
| Plasma glucose and hormones involved in host metabolism, satiety and appetite | | | | | | | | | | | |
| Glucose  (mmol/L) | 4.81 ± 0.09 | 4.89 ± 0.07 | 5.01 ± 0.08 | 0.030 | 4.96 ± 0.06 | 4.90 ± 0.06 | 4.78 ± 0.05 | 0.019 | -0.266  (-0.44 to -0.09) | 0.003 | 0.075 |
| HbA1c  (mmol/mol) | 37.1 ± 0.6 | 34.8 ± 0.5 | 33.5 ± 0.5 | <0.001 | 36.8 ± 0.4 | 34.1 ± 0.4 | 33.1 ± 0.3 | <0.001 | 0.02  (-0.8 to 0.8) | 0.964 | <0.001 |
| Insulin  (pmol/L) | 9.33 ± 0.54 | 9.36 ± 0.63 | 9.80 ± 0.61 | 0.573 | 9.51 ± 0.56 | 9.48 ± 0.52 | 10.11 ± 0.58 | 0.400 | 0.16  (-1.35 to 1.67) | 0.836 | <0.001 |
| C-peptide  (pg/ml) | 2029 ± 99 | 2007 ± 107 | 1951 ± 99 | 0.297 | 1935 ± 84 | 1900 ± 72 | 1962 ± 91 | 0.918 | 31.5  (-182 to 245) | 0.771 | 0.001 |
| Active Ghrelin  (pg/ml) | 558 ± 44 | 537 ± 54 | 515 ± 52 | 0.291 | 501 ± 31 | 596 ± 45 | 534 ± 35 | 0.468 | 50.2  (-63 to 163) | 0.382 | 0.007 |
| Total Ghrelin  (pg/ml) | 372 ± 40 | 284 ± 42 | 284 ± 29 | 0.012 | 300 ± 29 | 213 ± 26 | 274 ± 29 | 0.129 | 41.5  (-18 to 101) | 0.169 | 0.017 |
| Active GLP-1  (pM) | 0.59 ± 0.08 | 0.84 ± 0.10 | 0.86 ± 0.06 | 0.259 | 0.48 ± 0.05 | 0.75 ± 0.09 | 0.57 ± 0.07 | 0.291 | -0.105  (-0.30 to 0.09) | 0.292 | 0.010 |
| Total GLP-1  (pM) | 11.2 ± 0.7 | 14.8 ± 1.3 | 12.0 ± 0.8 | 0.061 | 10.3 ± 0.5 | 13.1 ± 0.7 | 11.7 ± 0.9 | 0.109 | -0.086  (-2.1 to 2.0) | 0.934 | <0.001 |
| % Active GLP-1 | 4.1 ± 0.3 | 6.3 ± 0.7 | 5.2 ± 0.4 | 0.010 | 5.0 ± 0.3 | 5.2 ± 0.3 | 4.9 ± 0.4 | 0.795 | -0.70  (-1.9 to 0.5) | 0.268 | 0.012 |
| PYY  (pg/ml) | 41.2 ± 3.0 | 43.6 ± 2.8 | 38.8 ± 2.3 | 0.801 | 36.6 ± 1.9 | 39.2 ± 2.5 | 34.9 ± 1.7 | 0.173 | -3.00  (-7.4 to 1.4) | 0.183 | 0.016 |
| Leptin  (ng/ml) | 26.7 ± 2.9 | 24.4 ± 2.7 | 24.5 ± 2.7 | 0.150 | 21.7 ± 2.2 | 22.1 ± 2.2 | 20.9 ± 2.2 | 0.341 | 0.68  (-2.7 to 4.0) | 0.690 | 0.001 |

## **Table S5. Overview of vital signs.**

|  | Placebo (n = 48) | | | | *B. longum* APC1472 (n = 74) | | | | Placebo vs *B. Longum* APC1472 | | |
| --- | --- | --- | --- | --- | --- | --- | --- | --- | --- | --- | --- |
| Variable | Week 0 | Week 6 | Week 12 | P-value  Week 0-12 | Week 0 | Week 6 | Week 12 | P-value  Week 0-12 | Difference  (95% CI) | P-value | η2 |

| Vital signs | | | | | | | | | | | |
| --- | --- | --- | --- | --- | --- | --- | --- | --- | --- | --- | --- |
| Ear temperature | 36.1 ± 0.1 | 36.1 ± 0.1 | 36.0 ±0.0 | 0.043 | 36.1 ± 0.1 | 36.0 ± 0.1 | 36 ± 0.0 | 0.095 | 0.056  (-0.1 to 0.2) | 0.378 | 0.007 |
| Systolic blood pressure | 122.8 ± 2.0 | 125.2 ± 2.3 | 124.8 ± 2.3 | 0.237 | 126.9 ± 1.7 | 125.6 ± 1.8 | 125.8 ± 1.8 | 0.451 | -2.03  (-6.0 to 1.9) | 0.313 | 0.009 |
| Diastolic blood pressure | 80.9 ± 1.1 | 81.4 ± 1.4 | 81.4 ± 1.2 | 0.637 | 83.1 ± 1.0 | 81.8 ± 1.1 | 82.0 ± 1.1 | 0.185 | -0.98  (-3.6 to 1.6) | 0.453 | 0.005 |
| Resting heart rate | 67.3 ± 1.2 | 67.6 ± 1.2 | 71.0 ± 1.1 | <0.001 | 69.5 ± 1.1 | 70.0 ± 1.2 | 72.2 ± 1.3 | 0.006 | -0.34  (-3.1 to 2.4) | 0.808 | 0.001 |

##

## **Table S6. Overview faecal SCFAs quantification data.**

|  | **Placebo (n = 48)** | | | ***B. longum* APC1472 (n = 74)** | | | **Placebo vs *B. Longum* APC1472** | | |
| --- | --- | --- | --- | --- | --- | --- | --- | --- | --- |
| Variable | Week 0 | Week 12 | P-value  Week 0-12 | Week 0 | Week 12 | P-value  Week 0-12 | Difference  (95% CI) | P-value | η^2^ |
| Acetate  µmol/g wet mass | 24.5 ± 2.8 | 18.7 ± 1.2 | 0.084 | 22.5 ± 2.1 | 18.5 ± 1.5 | 0.084 | -0.39  (-4.4 to 3.6) | 0.847 | 0.001 |
| Propionate  µmol/g wet mass | 11.1 ± 1.1 | 9.4 ± 0.6 | 0.113 | 10.9 ± 1.0 | 9.3 ± 0.6 | 0.112 | -0.21  (-1.9 to 1.5) | 0.806 | 0.001 |
| Butyrate  µmol/g wet mass | 9.7 ± 0.8 | 10.1 ± 0.8 | 0.693 | 10.8 ± 0.8 | 10.1 ± 0.7 | 0.464 | -0.29  (-2.4 to 1.8) | 0.782 | 0.001 |
| Valerate  µmol/g wet mass | 1.49 ± 0.12 | 1.36 ± 0.13 | 0.492 | 2.02 ± 0.52 | 1.34 ± 0.10 | 0.205 | -0.035  (-0.37 to 0.30) | 0.837 | 0.001 |
| Isobutyrate  µmol/g wet mass | 1.17 ± 0.08 | 1.26 ± 0.10 | 0.411 | 1.88 ± 0.52 | 1.30 ± 0.08 | 0.276 | 0.045  (-0.21 to 0.30) | 0.726 | 0.001 |
| Isovalerate  µmol/g wet mass | 2.04 ± 0.14 | 2.15 ± 0.18 | 0.539 | 2.68 ± 0.52 | 2.21 ± 0.14 | 0.371 | 0.049  (-0.40 to 0.50) | 0.828 | 0.001 |

Table S7. Baseline characteristics of obese subjects in the placebo and treatment arms at visit 1 (screening visit). Abbreviations: BMI = Body-mass index; W/H ratio = waist-to-hip ratio.

| Variable | Placebo  (n=36, mean ± STD) | *B. longum* APC1472  (n=46, mean ± STD) |
| --- | --- | --- |
| Weight (kg) | 90.0 ± 11.7 | 91.7 ± 10.6 |
| BMI | 32.0 ± 1.5 | 31.9 ± 1.4 |
| W/H ratio | 0.97 ± 0.08 | 0.97 ± 0.08 |
| Age (years) | 46.1 ± 10.5 | 45.8 ± 9.8 |
| Height (m) | 1.67 ± 0.10 | 1.69 ± 0.09 |
| Sex (% no. of subject) | | |
| Male | 41.7 | 50 |
| Female | 58.3 | 50 |
| Race or ethnicity (% no. of subject) | | |
| Caucasian | 100 | 100 |
| Arabic | 0 | 0 |
| Socioeconomic status (% no. of subject) | | |
| Non-manual | 36.1 | 26.1 |
| Lower Professional | 22.2 | 19.6 |
| Manual skilled | 11.1 | 13.0 |
| Semi-skilled | 11.1 | 15.2 |
| Employers and managers | 8.3 | 2.2 |
| Own account workers | 5.6 | 13 |
| Higher Professional | 5.6 | 6.5 |
| All others gainfully occupied and unknown | 0 | 0 |
| Farmer | 0 | 2.2 |
| Unskilled | 0 | 2.2 |
| Smoking status (% no. of subject) | | |
| Non-smoker | 44.4 | 52.2 |
| Past smoker | 33.3 | 43.5 |
| Current smoker | 22.2 | 4.3 |
| Alcohol consumption (mean ± SEM) | | |
| Units per week | 5.2 ± 4.7 | 4.3 ± 3.6 |
| Currently on concomitant medical or nutritional supplements (% no. of subject) | | |
| Yes | 30.6 | 41.3 |
| No | 69.4 | 58.7 |
| Compliance (% product consumed) | | |
| Week 6 | 96.2 ± 9.4 | 97.8 ± 7.4 |
| Week 12 | 94.6 ± 14.7 | 96.7 ± 11.8 |

## **Table S8. Overview of obesity-subpopulation analysis on anthropomorphic data and hormones involved in host metabolism, satiety and appetite**.

|  | **Placebo (n = 36)** | | | | | | | ***B. longum* APC1472 (n = 46)** | | | | | | | **Placebo vs *B. Longum* APC1472** | | | | |
| --- | --- | --- | --- | --- | --- | --- | --- | --- | --- | --- | --- | --- | --- | --- | --- | --- | --- | --- | --- |
| Variable | Week 0 | Week 6 | | Week 12 | | P-value  Week 0-12 | | Week 0 | | Week 6 | Week 12 | | P-value  Week 0-12 | | Difference  (95% CI) | P-value | | η^2^ | |
| Anthropomorphic data | | | | | | | | | | | | | | | | | | | |
| Weight (kg) | 90.1 ± 1.9 | | 90.2 ± 1.9 | | 90.3 ± 2.0 | | 0.476 | 91.7 ± 1.6 | 91.8 ± 1.6 | | | 91.5 ± 1.6 | | 0.676 | -0.33  (-1.2 to 0.6) | | 0.471 | | 0.007 |
| BMI (kg/m^2^) | 32.1 ± 0.3 | | 32.1 ± 0.3 | | 32.1 ± 0.3 | | 0.450 | 31.9 ± 0.2 | 31.9 ± 0.2 | | | 31.9 ± 0.2 | | 0.740 | -0.12  (-0.4 to 0.2) | | 0.483 | | 0.006 |
| W-H ratio | 0.96 ± 0.01 | | 0.96 ± 0.01 | | 0.96 ± 0.01 | | 0.777 | 0.97 ± 0.01 | 0.97 ± 0.01 | | | 0.97 ± 0.01 | | 0.032 | -0.006  (-0.0 to 0.0) | | 0.298 | | 0.014 |
| Plasma glucose and hormones involved in host metabolism, satiety and appetite | | | | | | | | | | | | | | | | | | | |
| Glucose  (mmol/L) | 4.8 ± 0.1 | | 4.9 ± 0.1 | | 5.0 ± 0.1 | | 0.046 | 5.0 ± 0.1 | 5.0 ± 0.1 | | | 4.8 ± 0.1 | | 0.016 | -0.295  (-0.5 to -0.1) | | 0.007 | | 0.092 |
| HbA1c  (mmol/mol) | 37.4 ± 0.7 | | 35.2 ± 3.6 | | 33.6 ± 0.5 | | <0.001 | 37.5 ± 0.5 | 34.7 ± 0.5 | | | 33.8 ± 0.4 | | <0.001 | 0.15  (-0.8 to 1.1) | | 0.766 | | 0.001 |
| Insulin  (pmol/L) | 9.7 ± 0.7 | | 9.7 ± 0.8 | | 10.1 ± 0.7 | | 0.543 | 10.1 ± 0.8 | 10.2 ± 0.7 | | | 11.0 ± 0.8 | | 0.458 | 0.35  (-1.7 to 2.4) | | 0.730 | | 0.002 |
| C-peptide  (pg/ml) | 2080 ± 123 | | 2061 ± 123 | | 2029 ± 120 | | 0.509 | 2052 ± 118 | 2009 ± 93 | | | 2024 ± 136 | | 0.839 | -0.0  (-296 to 296) | | 1.000 | | <0.001 |
| Active Ghrelin  (pg/ml) | 534 ± 40 | | 522 ± 55 | | 456 ± 36 | | 0.149 | 493 ± 36 | 551 ± 52 | | | 563 ± 47 | | 0.224 | 126.5  (13 to 240) | | 0.030 | | 0.062 |
| Total Ghrelin  (pg/ml) | 329 ± 39 | | 260 ± 39 | | 279 ± 29 | | 0.154 | 305 ± 38 | 248 ± 37 | | | 301 ± 40 | | 0.743 | 41.3  (-33 to 115) | | 0.269 | | 0.016 |
| Active GLP-1  (pM) | 0.58 ± 0.09 | | 0.83 ± 0.11 | | 0.71 ± 0.09 | | 0.282 | 0.52 ± 0.08 | 0.78 ± 0.12 | | | 0.56 ± 0.07 | | 0.647 | -0.152  (-0.38 to 0.07) | | 0.185 | | 0.025 |
| Total GLP-1  (pM) | 11.6 ± 0.8 | | 15.1 ± 1.5 | | 12.4 ± 1.0 | | 0.022 | 10.9 ± 0.7 | 13.2 ± 1.0 | | | 12.1 ± 1.2 | | 0.262 | 0.08  (-2.7 to 2.6) | | 0.954 | | <0.001 |
| PYY  (pg/ml) | 39.3 ± 2.8 | | 43.9 ± 3.3 | | 40.2 ± 2.8 | | 0.758 | 36.0 ± 2.3 | 39.9 ± 3.7 | | | 34.4 ± 2.3 | | 0.593 | -3.85  (-9.8 to 2.1) | | 0.199 | | 0.022 |
| Leptin  (ng/ml) | 28.3 ± 3.5 | | 26.3 ± 3.3 | | 26.8 ± 3.2 | | 0.250 | 23.6 ± 3.1 | 22.9 ± 2.9 | | | 21.1 ± 3.0 | | 0.202 | -0.42  (-4.7 to 3.8) | | 0.844 | | 0.001 |

## **Table S9. Overview of obesity-subpopulation analysis on vital signs, lipid, and inflammatory profiles.**

|  | **Placebo (n = 36)** | | | | | | | ***B. longum* APC1472 (n = 46)** | | | | | | | **Placebo vs *B. Longum* APC1472** | | | | |
| --- | --- | --- | --- | --- | --- | --- | --- | --- | --- | --- | --- | --- | --- | --- | --- | --- | --- | --- | --- |
| Variable | Week 0 | Week 6 | | Week 12 | | P-value  Week 0-12 | | Week 0 | | Week 6 | Week 12 | | P-value  Week 0-12 | | Difference  (95% CI) | P-value | | η^2^ | |
| Vital signs | | | | | | | | | | | | | | | | | | | |
| Ear temperature | 36.1 ± 0.1 | | 36.1 ± 0.1 | | 36.0 ± 0.01 | | 0.297 | 36.2 ± 0.1 | 36.1 ± 0.1 | | | 36.0 ± 0.01 | | 0.083 | 0.012  (-0.1 to 0.2) | | 0.868 | | <0.001 |
| Systolic blood pressure | 123.7 ± 2.4 | | 127.0 ± 2.6 | | 126.7 ± 2.7 | | 0.115 | 128.7 ± 2.3 | 128.1 ± 2.3 | | | 127.3 ± 2.3 | | 0.475 | 3.03  (-8.1 to 2.0) | | 0.237 | | 0.018 |
| Diastolic blood pressure | 81.4 ± 1.2 | | 81.5 ± 1.6 | | 82.2 ± 1.3 | | 0.495 | 84.5 ± 1.4 | 83.6 ± 1.3 | | | 83.0 ± 1.4 | | 0.233 | -1.25  (-4.5 to 2.0) | | 0.442 | | 0.008 |
| Resting heart rate | 67.9 ± 1.5 | | 67.1 ± 1.5 | | 71.4 ± 1.3 | | 0.007 | 69.6 ± 1.5 | 69.3 ± 1.4 | | | 71.9 ± 1.7 | | 0.083 | -0.72  (-4.1 to 2.7) | | 0.672 | | 0.002 |
| Lipid profile | | | | | | | | | | | | | | | | | | | |
| Cholesterol  (mmol/L) | 5.5 ± 0.1 | | 5.5 ± 0.1 | | 5.4 ± 0.18 | | 0.604 | 5.4 ± 0.1 | 5.4 ± 0.1 | | | 5.3 ± 0.12 | | 0.474 | -0.05  (-0.5 to 0.3) | | 0.790 | | 0.001 |
| Triglycerides  (mmol/L) | 1.4 ± 0.1 | | 1.5 ± 01 | | 1.4 ± 0.1 | | 0.493 | 1.5 ± 0.1 | 1.5 ± 0.1 | | | 1.5 ± 0.1 | | 0.781 | 0.10  (-0.2 to 0.3) | | 0.445 | | 0.007 |
| LDL  (mmol/L | 3.8 ± 0.1 | | 3.8 ± 0.1 | | 3.8 ± 0.2 | | 0.841 | 3.7 ± 0.1 | 3.7 ± 0.2 | | | 3.6 ± 0.1 | | 0.356 | -0.21  (-0.6 to 0.2) | | 0.267 | | 0.016 |
| HDL  (mmol/L) | 1.3 ± 0.0 | | 1.3 ± 0.0 | | 1.3 ± 0.0 | | 0.938 | 1.3 ± 0.1 | 1.3 ± 0.1 | | | 1.4 ± 0.1 | | 0.187 | 0.059  (-0.0 to 0.2) | | 0.324 | | 0.012 |
| Inflammatory profile | | | | | | | | | | | | | | | | | | | |
| IL-10  (pg/ml) | 0.46 ± 0.07 | | 0.49 ± 0.08 | | 0.45 ± 0.06 | | 0.726 | 0.38 ± 0.03 | 0.38 ± 0.04 | | | 0.40 ± 0.08 | | 0.727 | -0.015  (-0.22 to 0.19) | | 0.884 | | <0.001 |
| TNF-α  (pg/ml) | 1.20 ± 0.13 | | 1.25 ± 0.15 | | 1.07 ± 0.11 | | 0.414 | 0.94 ± 0.08 | 0.89 ± 0.08 | | | 0.90 ± 0.09 | | 0.808 | 0.001  (-0.15 to 0.16) | | 0.988 | | <0.001 |
| IFNγ  (pg/ml) | 10.3 ± 1.7 | | 10.1 ± 1.4 | | 10.8 ± 2.0 | | 0.804 | 6.9 ± 1.1 | 5.4 ± 0.6 | | | 10.9 ± 4.8 | | 0.453 | -0.58  (-12.7 to 11.6) | | 0.925 | | <0.001 |

## **Table S10. Overview of obesity-subpopulation analysis on satiety, mood, perceived stress and cortisol awakening response data.**

|  | **Placebo (n = 36)** | | | | ***B. longum* APC1472 (n = 46)** | | | | **Placebo vs *B. Longum* APC1472** | | |
| --- | --- | --- | --- | --- | --- | --- | --- | --- | --- | --- | --- |
| Variable | Week 0 | Week 6 | Week 12 | P-value  Week 0-12 | Week 0 | Week 6 | Week 12 | P-value  Week 0-12 | Difference  (95% CI) | P-value | η^2^ |
| Questionnaire data | | | | | | | | | | | |
| Perceived stress  (Cohens PSS) | 11.0 ± 0.9 | 10.6 ± 0.9 | 10.3 ± 1.0 | 0.365 | 12.7 ± 0.8 | 10.8 ± 0.9 | 10.3 ± 1.0 | 0.006 | -1.26  (-3.6 to 1.0) | 0.280 | 0.015 |
| Depression  (HADS) | 2.9 ± 0.4 | 2.6 ± 0.4 | 2.1 ± 0.4 | 0.067 | 2.8 ± 0.4 | 2.6 ± 0.5 | 2.2 ± 0.4 | 0.081 | 0.09  (-0.8 to 1.0) | 0.838 | 0.001 |
| Anxiety  (HADS) | 4.9 ± 0.5 | 4.5 ± 0.6 | 4.4 ± 0.6 | 0.278 | 4.8 ± 0.5 | 4.8 ± 0.6 | 4.3 ± 0.6 | 0.199 | -0.002  (-1.3 to 1.3) | 0.997 | <0.001 |
| Hunger/Satiety | 5.1 ± 0.5 | 4.9 ± 0.4 | 6.2 ± 0.5 | 0.098 | 5.3 ± 0.4 | 5.9 ± 0.4 | 5.8 ± 0.4 | 0.207 | -0.42  (-1.6 to 0.7) | 0.470 | 0.007 |
| Cortisol awakening response | | | | | | | | | | | |
| AUCi  (nmol/L) | 2.5 ± 9.4 | / | 15.6 ± 5.4 | 0.393 | 2.65 ± 3.94 | / | 6.4 ± 6.7 | 0.531 | -13.57  (-26.5 to -0.6) | 0.041 | 0.080 |
| AUC  (nmol/L) | 31.7 ± 3.1 | / | 38.3 ± 4.7 | 0.115 | 45.1 ± 5.2 | / | 43.6 ± 4.0 | 0.625 | -1.67  (-14.7 to 11.4) | 0.797 | 0.001 |
| Average  (nmol/L) | 8.2 ± 0.7 | / | 10.1 ± 1.2 | 0.158 | 11.8 ± 1.4 | / | 10.2 ± 1.0 | 0.418 | -0.81  (-4.0 to 2.3) | 0.607 | 0.004 |

## **Table S11. Overview of obesity-subpopulation analysis on faecal SCFAs quantification data.**

|  | **Placebo (n = 36)** | | | ***B. longum* APC1472 (n = 46)** | | | **Placebo vs *B. Longum* APC1472** | | |
| --- | --- | --- | --- | --- | --- | --- | --- | --- | --- |
| Variable | Week 0 | Week 12 | P-value  Week 0-12 | Week 0 | Week 12 | P-value  Week 0-12 | Difference  (95% CI) | P-value | η^2^ |
| Acetate  µmol/g wet mass | 25.9 ± 3.7 | 19.2 ± 1.3 | 0.108 | 24.6 ± 3.3 | 19.5 ± 2.1 | 0.162 | -0.04  (-5.1 to 5.1) | 0.987 | 0.001 |
| Propionate  µmol/g wet mass | 11.6 ± 1.3 | 10.3 ± 0.7 | 0.296 | 11.3 ± 1.2 | 10.4 ± 0.9 | 0.384 | 0.05  (-2.1 to 2.2) | 0.960 | 0.001 |
| Butyrate  µmol/g wet mass | 9.7 ± 1.0 | 10.3 ± 0.7 | 0.591 | 11.3 ± 1.1 | 10.9 ± 0.9 | 0.735 | 0.21  (-2.1 to 2.5) | 0.856 | 0.001 |
| Valerate  µmol/g wet mass | 1.43 ± 0.13 | 1.46 ± 0.17 | 0.910 | 1.94 ± 0.61 | 1.48 ± 0.15 | 0.441 | -0.010  (-0.47 to 0.45) | 0.965 | 0.001 |
| Isobutyrate  µmol/g wet mass | 1.13 ± 0.10 | 1.26 ± 0.13 | 0.288 | 1.86 ± 0.60 | 1.34 ± 0.12 | 0.373 | 0.044  (-0.30 to 0.39) | 0.798 | 0.001 |
| Isovalerate  µmol/g wet mass | 1.96 ± 0.16 | 2.12 ± 0.22 | 0.431 | 2.73 ± 0.61 | 2.24 ± 0.20 | 0.406 | 0.042  (-0.54 to 0.63) | 0.887 | 0.001 |
